# A novel recognition site for polyubiquitin and ubiquitin-like signals in an unexpected region of proteasomal subunit Rpn1

**DOI:** 10.1016/j.jbc.2021.101052

**Published:** 2021-08-06

**Authors:** Andrew J. Boughton, Leonard Liu, Tali Lavy, Oded Kleifeld, David Fushman

**Affiliations:** 1 Department of Chemistry and Biochemistry, Center for Biomolecular Structure and Organization, University of Maryland, College Park, Maryland, USA; 2 Faculty of Biology, Technion-Israel Institute of Technology, Technion City, Haifa, Israel

**Keywords:** proteasome, ubiquitin, UBL domain, K11-linked polyubiquitin, K48-linked polyubiquitin, Rad23, Dsk2, Ubp6, Rpn1, Bpa, *p*-benzoyl-l-phenylalanine, CP, core particle, CSP, chemical shift perturbation, GuHCl, guanidinium chloride, MTSL, (1-oxyl-2,2,5,5-tetramethyl-3-pyrroline-3-methyl) methanesulfonate, NT, *N*-terminal to *T*oroid, PC, proteasome/cyclosome, PDB, Protein Data Bank, polyUb, polymeric Ub, PRE, paramagnetic relaxation enhancement, RP, regulatory particle, Rpn1^214–355^, Rpn1 encompassing residues 214 to 355, TCEP, tris(2-carboxyethyl)phosphine, Ub, ubiquitin, UBA, Ub-associated, UBL, Ub-like, UPS, ubiquitin–proteasome system

## Abstract

The ubiquitin (Ub)–proteasome system is the primary mechanism for maintaining protein homeostasis in eukaryotes, yet the underlying signaling events and specificities of its components are poorly understood. Proteins destined for degradation are tagged with covalently linked polymeric Ub chains and subsequently delivered to the proteasome, often with the assistance of shuttle proteins that contain Ub-like domains. This degradation pathway is riddled with apparent redundancy—in the form of numerous polyubiquitin chains of various lengths and distinct architectures, multiple shuttle proteins, and at least three proteasomal receptors. Moreover, the largest proteasomal receptor, Rpn1, contains one known binding site for polyubiquitin and shuttle proteins, although several studies have recently proposed the existence of an additional uncharacterized site. Here, using a combination of NMR spectroscopy, photocrosslinking, mass spectrometry, and mutagenesis, we show that Rpn1 does indeed contain another recognition site that exhibits affinities and binding preferences for polyubiquitin and Ub-like signals comparable to those of the known binding site in Rpn1. Surprisingly, this novel site is situated in the N-terminal section of Rpn1, a region previously surmised to be devoid of functionality. We identified a stretch of adjacent helices as the location of this previously uncharacterized binding site, whose spatial proximity and similar properties to the known binding site in Rpn1 suggest the possibility of multivalent signal recognition across the solvent-exposed surface of Rpn1. These findings offer new mechanistic insights into signal recognition processes that are at the core of the Ub–proteasome system.

Eukaryotic protein turnover relies on the careful coordination of substrates, ubiquitin (Ub), and the proteasome—collectively known as the ubiquitin–proteasome system (UPS)—and is essential for cell survivability (1). UPS-mediated protein degradation occurs *via* substrate conjugation to specific polymeric Ub (polyUb) chains through an ATP-dependent enzymatic process, after which the 26S proteasome recognizes the polyUb tag and subsequently degrades the substrate. Although many UPS components implicated in substrate conjugation are relatively well characterized, the interactions and specificity of proteasomal signal recognition are more ambiguous.

There are two distinct modes of signal recognition by the 26S proteasome—direct and indirect. In the direct mode, a polyUb tag that is conjugated to a substrate is directly recognized by the proteasome (2, 3, 4, 5, 6, 7). Alternatively, shuttle proteins that contain Ub-like (UBL) and Ub-associated (UBA) domains indirectly escort polyubiquitinated substrates to the proteasome, whereby the UBA domain binds to polyUb (8, 9) and the UBL domain binds to the proteasome (5, 6, 7, 10, 11, 12, 13, 14, 15). Of these UBL–UBA shuttle proteins, Rad23/hHR23 (yeast/human) and Dsk2/hPLIC-1/Ubiquilin-1 are the most prominent (9, 16). Other extrinsic factors may also participate in this pathway, such as purported shuttle protein Ddi1/hDDI1 (14), which contains a UBA domain (absent in mammals) and an atypical UBL domain (12), and Ubp6/hUSP14 (17), a transient proteasome-associated deubiquitinase with a UBL domain but no UBA domain.

Just as there are multiple signals that target substrates for degradation, there are also multiple receptors on the proteasome. The 26S proteasome is a massive 2.5 MDa complex that is typically composed of three multisubunit subassemblies: two 19S regulatory particles (RPs) and one 20S core particle (CP). Signals are recognized by the RP, and substrates are subsequently fed into the proteolytically active CP to be degraded (18). The RP contains three known receptors—Rpn1/PSMD2, Rpn10/S5a, and Rpn13/ADRM1—all of which recognize polyUb and the UBL domains of shuttle proteins, to varying extents (2, 3, 4, 5, 6, 7, 10, 11, 12, 13, 14, 15).

Of these RP receptors, Rpn1 is the largest (∼110 kDa) and least characterized. Although the structure of Rpn1 has not yet been determined to high resolution, it is predicted to contain 9 to 11 helix–turn–helix proteasome/cyclosome (PC) repeats, each up to 40 residues long (19, 20). The PC repeats are clustered into the central portion of Rpn1, flanked by flexible termini (Fig. 1*A*). Several studies have shown that the PC repeat region harbors a recognition site for polyUb and UBL signals (7, 10, 11, 14); this site was subsequently mapped to three helices in the solvent-exposed segment of the PC repeat region, known as the T1 site (6, 15).

**Figure 1:**
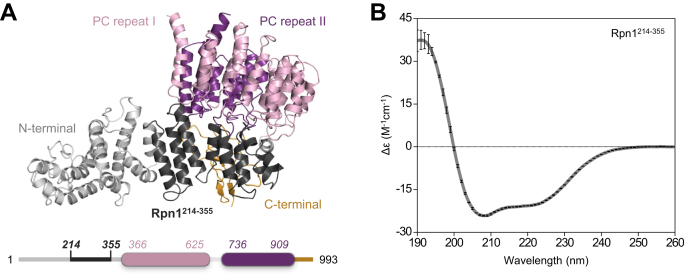
**Structural properties of Rpn1.***A*, structure of Rpn1 (Protein Data Bank: 5MPC); the C-terminal region is *gold*, the toroidal PC repeat regions are *pink* and *purple*, the N-terminal region is *gray*, and the region encompassing residues 214 to 355 is *black*. A schematic of the sequence of Rpn1 is shown below, with residue numbers and the aforementioned coloring scheme. *B*, CD spectrum of Rpn1^214–355^ at a concentration of 0.2 mg/ml. Data were recorded in triplicate, with error bars corresponding to standard deviations in ellipticity across the three datasets. PC, proteasome/cyclosome.

Interestingly, Rpn1 is always present in the proteasome assembly, while a significant portion of active proteasomes function without Rpn10 or Rpn13 (21). Moreover, Rpn1 contains several potential polyUb/UBL recognition motifs (PC repeats (6, 15)), whereas Rpn10 (UIM domain (2, 22)) and Rpn13 (Pru domain (5)) each have one. Even though only one recognition site for proteasomal signals has been identified in Rpn1 so far, multiple studies have recently suggested the presence of additional sites (6, 11, 23, 24).

Abnormalities in the UPS are associated with cancers, neurodegenerative diseases, metabolic disorders, muscular dystrophies, and more (25). Thus, the relationship between Ub and the proteasome has been intensely studied. Even so, the requirement for multiple degradation signals, recognition modes, and proteasomal receptors remains unexplained. Here, we show that an N-terminal fragment of Rpn1 encompassing residues 214 to 355 (Rpn1^214–355^) contains a recognition site for signals, such as Ub, polyUb, and UBL domains. This result is surprising, as Rpn1 is thought to interact with proteasomal signals through its PC repeats (6, 15), none of which are present in Rpn1^214–355^ (Fig. 1*A*). Our binding assays demonstrated that several of these interactions exhibit physiologically relevant binding affinity. Using a combination of NMR spectroscopy, photocrosslinking, mass spectrometry, and mutagenesis, the location of this binding site was ultimately narrowed down to a small region of adjacent helices, whose global positioning suggests the possibility of multisite recognition events across Rpn1.

## Results

### Rpn1^214–355^ associates with Ub and is predominantly helical

Given the previous suggestions that Rpn1 may contain multiple recognition sites (6, 11, 23, 24), we screened several isolated fragments of Rpn1 for binding to monoUb. This analysis identified one construct, Rpn1^214–355^, which elicited perturbations in the NMR signals of ^15^N-monoUb (Fig. S1*A*). Rpn1^214–355^ also produced perturbations in the NMR signals of ^15^N-Rub1 (Fig. S1*B*), a UBL protein with an identical tertiary fold and 53% sequence identity to Ub. Therefore, we further investigated Rpn1^214–355^. This construct could not be expressed as a soluble protein; instead, Rpn1^214–355^ was purified from the insoluble lysate fraction using urea. The resulting protein migrated as expected on SDS-PAGE (Fig. S2*A*) and exhibited the correct mass (Fig. S2*B*).

CD spectroscopy was used to verify that Rpn1^214–355^ refolded after removing urea. The CD spectrum of Rpn1^214–355^ exhibited clear minima at 222 and 208 nm (Fig. 1*B*), which are distinctly helical characteristics (26). Rpn1^214–355^ was predicted to be at least ∼75% helical based on existing cryogenic electron microscopy structural models, with a small percentage of turns and unfolded regions (Table 1). Indeed, deconvolution of the experimental ellipticity data showed that Rpn1^214–355^ was upward of ∼85% helical (Table 1). These results indicated that Rpn1^214–355^ was folded and displayed the expected structural characteristics, despite being an isolated fragment of a larger protein. Moreover, the ratio of ellipticity at 222 and 208 nm (Δε_222_/Δε_208_) was 0.84; Δε_222_/Δε_208_ values below 0.9 typically suggest the presence of long and isolated helices. Deconvolution results corroborated this observation, with an estimated average helix length of ∼16 residues (Table S1). Overall, these data agree with structural models of Rpn1, which contains numerous long helices that align to form a toroidal structure (19, 20).

**Table 1:** Secondary structure characterization of Rpn1^214–355^

Secondary structure characterization of Rpn1^214–355^ from PDB: 5MPC^a^^,^^b^				
Method	Helix (%)	Strand (%)	Turn (%)	Unordered (%)
STRIDE^c^	76.8	0.0	14.8	8.5
DSSP^d^	71.8	0.0	12.7	15.5

Secondary structure prediction of Rpn1^214–355^ from experimental CD data^e^						
Method	Regular helix (%)	Distorted helix (%)	Regular strand (%)	Distorted strand (%)	Turn (%)	Unordered (%)
CONTINLL^f^	74.3	24.6	0.0	1.1	0.0	0.0
CDSSTR^g^	64.4	19.5	2.0	2.3	5.6	5.5

a The 2Struc Secondary Structure Server (55) was used for secondary structure analysis from a PDB file.

b The structure of Rpn1 (PDB: 5MPC) was described previously (56).

c The STRuctural IDEntification method (57) uses hydrogen bond energies and phi–psi torsion angles to identify secondary structure.

d The Dictionary of Secondary Structure of Proteins (58) uses hydrogen bond energies to identify secondary structure.

e The DICHROWEB server (41) was used to analyze CD data.

f The CONTINLL deconvolution method was described previously (42). The normalized RMSD for this method was 0.062. Deconvolution utilized reference protein datasets 4, 7, SP175, and SMP180 (44, 45, 46). Percent values for each type of secondary structure are averages across all reference sets.

g The CDSSTR deconvolution method was described previously (43). The normalized RMSD for this method was 0.001. Deconvolution utilized reference protein datasets 4, 7, SP175, and SMP180 (44, 45, 46). Percent values for each type of secondary structure are averages across all reference sets.

### Rpn1^214–355^ crosslinks with Ub and polyUb

**Figure 2:**
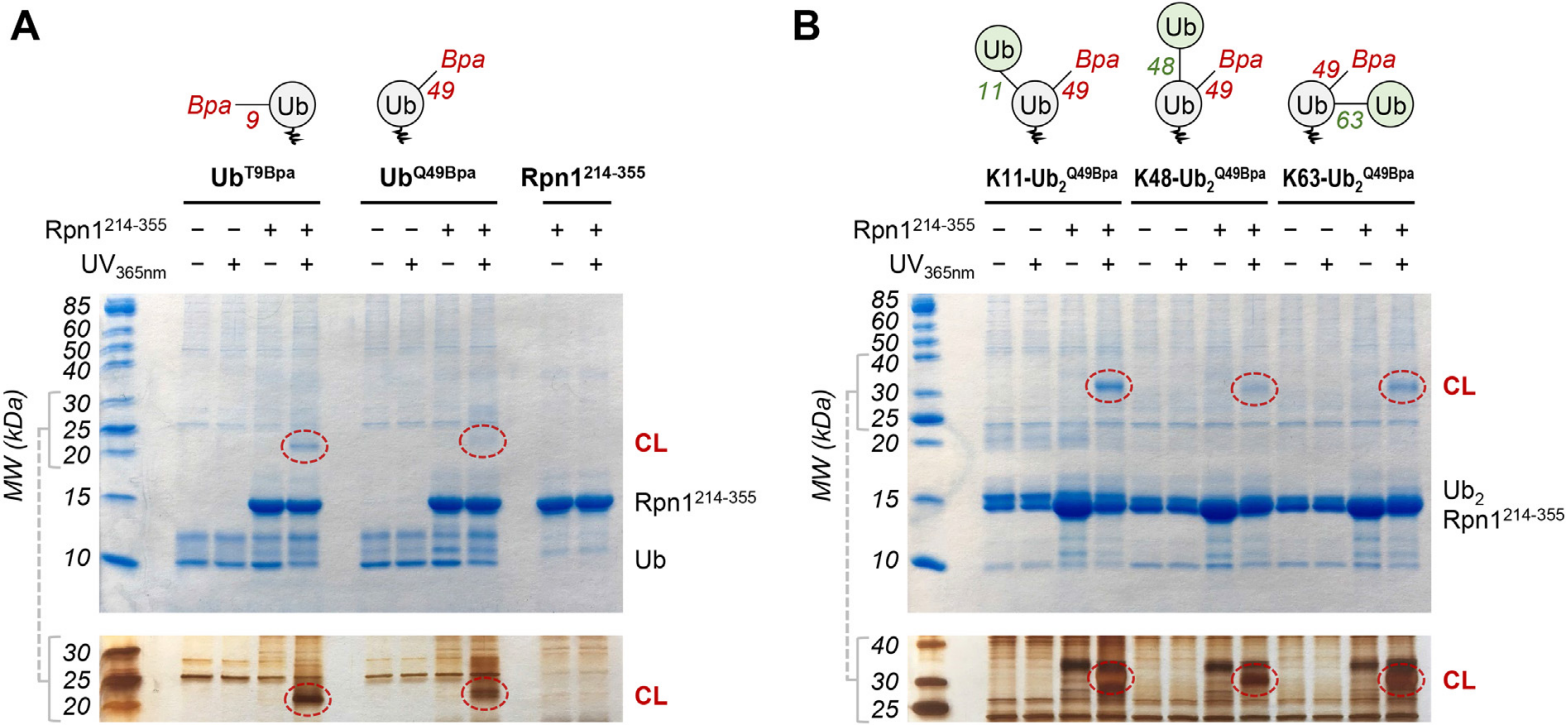
**Rpn1**^**214–355**^**crosslinks with Ub and Ub**_**2**_**.** SDS-PAGE gels showing the results of exposure to UV_365nm_ irradiation of (*A*) Bpa-containing Ub moieties and/or Rpn1^214–355^; (*B*) Bpa-containing Ub_2_ moieties and Rpn1^214–355^. *A* and *B*, the moiety written in *bold* above the gel is always present in the respective gel lanes, whereas addition of Rpn1^214–355^ or exposure to UV_365nm_ irradiation is indicated by *plus/minus* symbols. Both Coomassie blue (*top*) and silver (*bottom*) staining were performed. Crosslinked products (CL) are indicated by *red circles*. A schematic of each Ub or Ub_2_ moiety is shown at the *top*, wherein Bpa is *red*, the Bpa-attached Ub is *gray*, the distal Ub (if present) is *green*, and the residue number of the linked lysine (if present) is *green*. Bpa, *p*-benzoyl-l-phenylalanine; Ub, ubiquitin.

To examine the extent of Rpn1^214–355^ interaction with proteasomal signals, photocrosslinking reactions were performed with Ub moieties that contained *p*-benzoyl-l-phenylalanine (Bpa). Photoactivatable Bpa was specifically incorporated as a genetically encoded unnatural amino acid at either position 9 (Ub^T9Bpa^) or position 49 (Ub^Q49Bpa^) in Ub (see schematic in Fig. 2*A*), as these locations were successful in previous studies (27). Significant crosslinking was observed after subjecting mixtures of Rpn1^214–355^ and Ub^T9Bpa^ or Ub^Q49Bpa^ to UV_365nm_ irradiation (Fig. 2*A*), indicative of binding between Rpn1^214–355^ and Ub. It is important to note that this product must be the result of intermolecular Ub–Rpn1^214–355^ crosslinking, as reactions containing only Ub or Rpn1^214–355^ did not show any evidence of crosslinking (Fig. 2*A*).

Encouraged by these results, we performed additional photocrosslinking experiments using Bpa-containing K11-linked Ub_2_ (K11-Ub_2_^Q49Bpa^), K48-linked Ub_2_ (K48-Ub_2_^Q49Bpa^), and K63-linked Ub_2_ (K63-Ub_2_^Q49Bpa^); in these dimers, Bpa was always incorporated at position 49 in the proximal (lysine-donating) Ub (see schematic in Fig. 2*B*). Crosslinking with Rpn1^214–355^ was observed for all three dimers (Fig. 2*B*), and band intensities were stronger than those seen in reactions with monoUb; this is not surprising, as other Rpn1 constructs have shown greater affinity for Ub_2_ than for Ub (7, 28). Notably, the reaction with K11-Ub_2_^Q49Bpa^ exhibited the largest amount of crosslinked product, indicating that Rpn1^214–355^ may preferentially associate with K11-linked polyUb over K48-linked polyUb. A similar preference for K11-linked polyUb was observed with Rpn1^391–642^ (24, 28), a region that includes the T1 site in Rpn1 (6, 7). Based on all these observations, it is evident that the N-terminal section of Rpn1 (encompassing residues 214–355) possesses a recognition site for Ub and polyUb signals, even though it does not contain any of the hallmark PC repeats.

### Rpn1^214–355^ binds polyUb and UBL moieties with physiologically relevant affinities

NMR titration experiments were utilized to further confirm and quantify the affinity of Rpn1^214–355^ for various proteasomal signals, with a focus on polyUb moieties and the UBL domains of proteasome-associated proteins.

**Figure 3:**
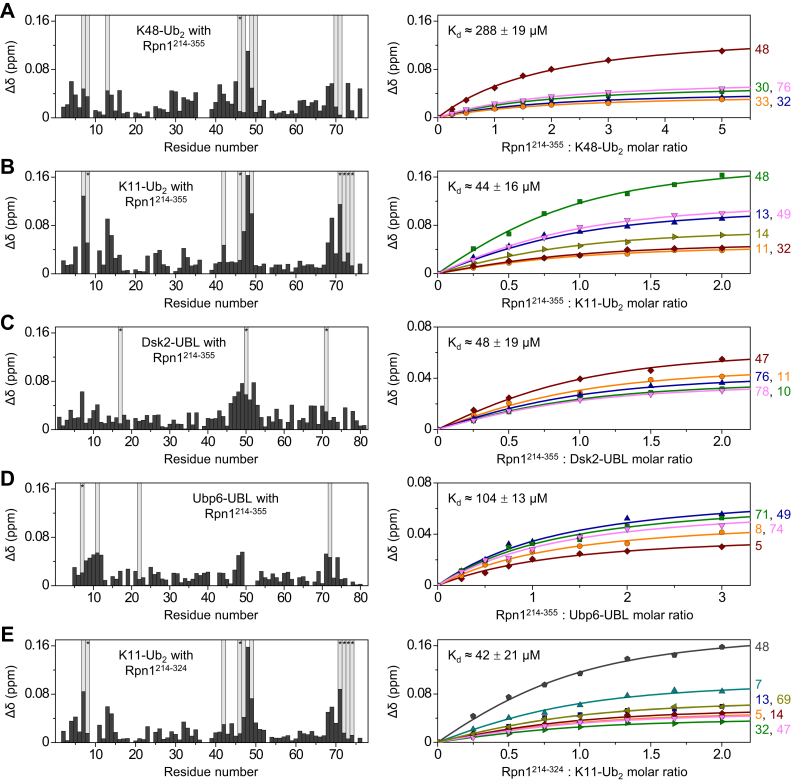
**Rpn1**^**214–355**^**binds Ub**_**2**_**and Ub-like species.***A*–*D*, NMR titration data for Rpn1^214–355^ binding to (*A*) ^15^N-^d^K48-Ub_2_; (*B*) ^15^N-^d^K11-Ub_2_; (*C*) ^15^N-Dsk2-UBL; and (*D*) ^15^N-Ubp6-UBL. *E*, NMR titration data for Rpn1^214–324^ binding to ^15^N-^d^K11-Ub_2_. *Left*, residue-specific CSPs (Δδ, *black bars*) for each protein at the endpoint of titration with each Rpn1 construct. *Light gray bars* indicate residues that exhibited a signal intensity ratio less than the mean minus standard deviation at an equimolar Rpn1:^15^N-protein ratio, with *asterisks* denoting residues whose signal completely disappeared. *Right*, titration curves show CSPs (symbols) as a function of the Rpn1:^15^N-protein molar ratio; the lines represent the fit to a single-site binding model. Residue numbers are indicated to the right of titration curves. In all five titrations, the initial concentration of the ^15^N-enriched protein was 150 μM. CSP, chemical shift perturbation; Ub, ubiquitin.

Upon addition of Rpn1^214–355^, the NMR spectra of K48-linked Ub_2_ with the distal (lysine-accepting) Ub ^15^N-enriched (^15^N-^d^K48-Ub_2_) displayed significant signal shifts and attenuations—characteristic indicators of binding (Fig. S3*A*). These signal shifts, quantified on a per-residue basis as chemical shift perturbations (CSPs), were prevalent in and around the hydrophobic surface patch residues L8, I44, and V70 (the typical ligand-binding surface of Ub (29)) (Fig. 3*A*). A comparable CSP profile was observed for ^15^N-monoUb (Fig. S1*A*), suggesting that the same residues are involved in Rpn1^214–355^ binding. The dissociation constant (*K*_*d*_) for the interaction of ^15^N-^d^K48-Ub_2_ and Rpn1^214–355^ was 288 ± 19 μM.

A similar effect was observed in the NMR spectra of K11-linked Ub_2_ with the distal Ub ^15^N-enriched (^15^N-^d^K11-Ub_2_) after adding Rpn1^214–355^ (Fig. S3*B*), with significant CSPs corresponding to the hydrophobic patch region (Fig. 3*B*). Interestingly, binding was notably stronger than for ^15^N-^d^K48-Ub_2_, with a *K*_*d*_ of 44 ± 16 μM. It is worth reiterating that Rpn1^391–642^ (which contains the T1 site) also exhibited stronger affinity for K11-linked polyUb than for K48-linked polyUb (Table 2) (28). Collectively, the interaction between Rpn1^214–355^ and K11-linked Ub_2_ or K48-linked Ub_2_ was observed for both the distal Ub (NMR experiments) and the proximal Ub (photocrosslinking experiments).

**Table 2:** Comparison of *K*_*d*_ values for polyUb and UBLs binding to Rpn1^214–355^ and Rpn1^391–642^

*K*_*d*_ (μM)	^d^K48-Ub_2_	^d^K11-Ub_2_	Dsk2-UBL	Ubp6-UBL	Rad23-UBL	Ddi1-UBL
Rpn1^214–355^ (NT site)	288 ± 19	44 ± 16	48 ± 19	104 ± 13	Tight binding (attenuations)	No binding
Rpn1^391–642^ (T1 site)	123 ± 34 (28)	28 ± 6 (28)	22 ± 12 (7)	40 ± 31 (7)	Tight binding (24) (attenuations)	No binding (24)

Ub_2_ and UBL proteins were ^15^N enriched, whereas Rpn1 constructs were at natural abundance. Error values represent the standard deviation among several amino acid residues.

We next characterized Rpn1^214–355^ interactions with UBL domains from the aforementioned proteasome-associated proteins. Substantial perturbations were observed in the NMR spectra of ^15^N-Dsk2-UBL and ^15^N-Ubp6-UBL upon addition of Rpn1^214–355^ (Fig. S4), with *K*_*d*_ values of 48 ± 19 μM for Dsk2-UBL and 104 ± 13 μM for Ubp6-UBL (Fig. 3, *C* and *D*). Thus, the UBL domains of the shuttle protein Dsk2 and the deubiquitinase Ubp6 bind Rpn1^214–355^ with physiologically relevant affinities. By comparing the binding properties of Rpn1^214–355^ and Rpn1^391–642^, it is evident that Rpn1^214–355^ recognizes all the same proteasomal signals as Rpn1^391–642^, but with roughly two times larger *K*_*d*_ values (Table 2).

Rpn1^214–355^ appeared to exhibit strong affinity for the UBL domain of the shuttle protein Rad23, as the majority of ^15^N-Rad23-UBL NMR signals completely attenuated before an equimolar ^15^N-Rad23-UBL:Rpn1^214–355^ ratio was reached (Fig. S5); a similar phenomenon was observed for Rad23-UBL binding to other regions of Rpn1 (7, 24). This effect may be caused by slow or intermediate exchange on the NMR timescale, although the widespread disappearance of signals across the entire UBL domain is more indicative of signal broadening related to an increase in molecular weight (Fig. S5), perhaps as a result of oligomerization of the Rad23-UBL:Rpn1^214–355^ complex upon binding. Notably, the reported *K*_*d*_ for Rad23-UBL binding to the T1 site in Rpn1 is ∼64 nM (6). The disappearance of ^15^N-Rad23-UBL NMR signals prevented us from quantifying the affinity for Rpn1^214–355^, although we suspect that the interaction between Rad23-UBL and Rpn1^214–355^ is tight because of the similarities among observations from equivalent experiments with Rpn1^391–642^.

Meanwhile, the UBL domain of purported proteasomal shuttle Ddi1 did not show detectable interaction with Rpn1^214–355^ (Fig. S6), just as with several other Rpn1 constructs (24). Overall, these data indicate that the novel recognition site in Rpn1^214–355^ exhibits similar characteristics to the analogous site in Rpn1^391–642^, albeit with slightly weaker affinity for proteasomal signals.

### Spin-labeling experiments narrow down the putative recognition region in Rpn1^214–355^

Although Rpn1^214–355^ encompasses less than 15% of full-length Rpn1, we aimed to pinpoint the location of this novel recognition site even further. Unfortunately, the instability and low yield of Rpn1^214–355^ prevented us from performing NMR experiments with isotopically enriched Rpn1^214–355^ to identify residues involved in binding. Therefore, an alternative approach—site-directed paramagnetic spin labeling—was utilized to locate the binding site in Rpn1^214–355^.

**Figure 4:**
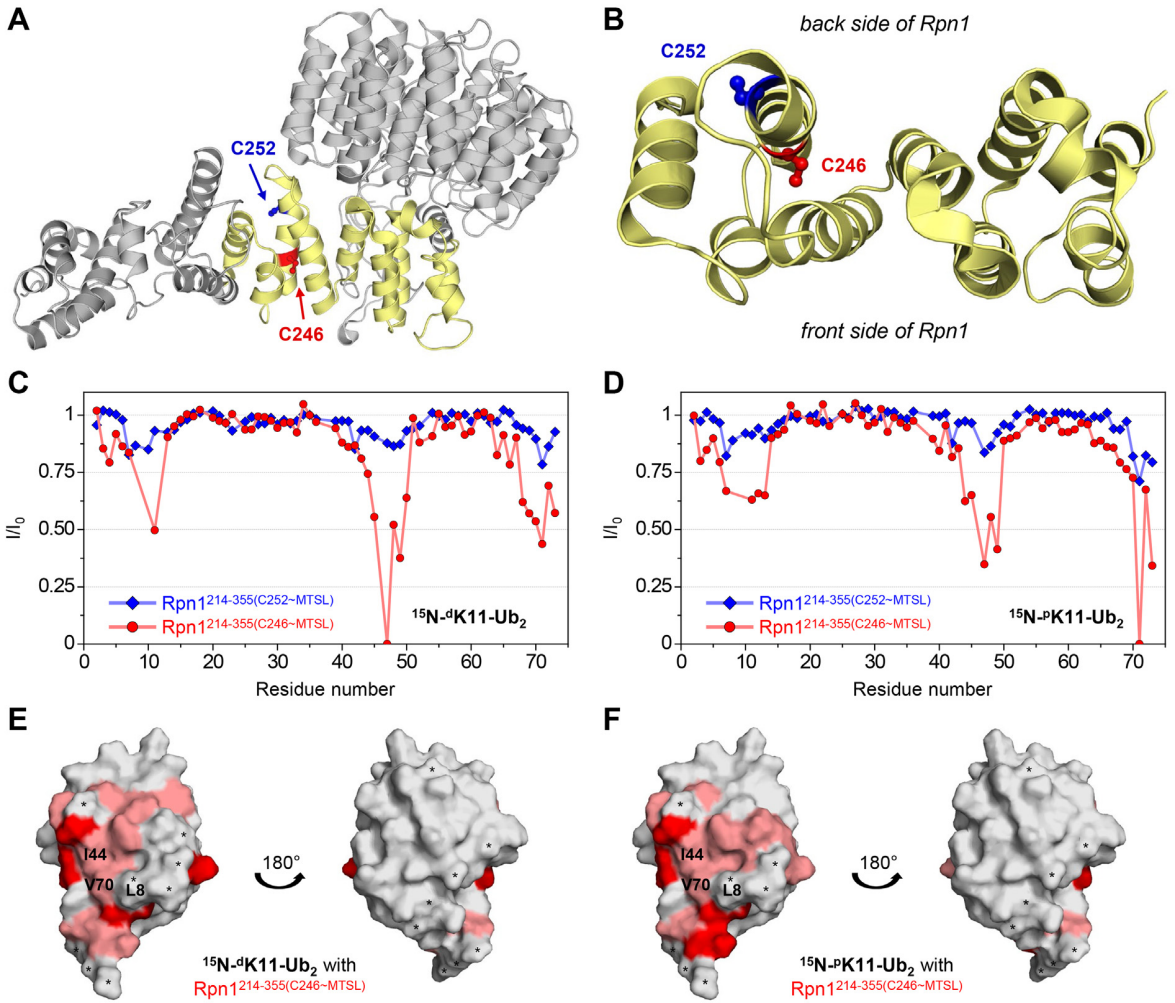
**Rpn1**^**214–355**^**binds the distal and proximal domains of K11-linked Ub**_**2**_**through analogous modes.***A*, structure of Rpn1 (Protein Data Bank: 4CR2), where the region encompassing residues 214 to 355 is *yellow*. In this orientation, the back side of Rpn1 (contacting the ATPase ring) is behind the page, whereas the solvent-exposed front side of Rpn1 is sticking out of the page. The two cysteines in this region are shown as *red* (C246) and *blue* (C252) sticks. *B*, top–down view of (*A*), utilizing the same color scheme. In this orientation, the back side of Rpn1 is toward the top of the image, whereas the solvent-exposed front side of Rpn1 is toward the bottom. *C*, PREs (*I*/*I*_0_) in ^15^N-^d^K11-Ub_2_ when mixed with an equimolar amount (115 μM of each protein) of Rpn1^214–355(C246∼MTSL)^ (*red circles*) or Rpn1^214–355(C252∼MTSL)^ (*blue diamonds*). *D*, PREs (*I*/*I*_0_) in ^15^N-^p^K11-Ub_2_ when mixed with an equimolar amount (115 μM of each protein) of Rpn1^214–355(C246∼MTSL)^ (*red circles*) or Rpn1^214–355(C252∼MTSL)^ (*blue diamonds*). *E*, structure of Ub (PDB: 1D3Z), where residues that exhibited diminished *I*/*I*_0_ values in (*C*) are colored as follows: *I*/*I*_0_ < 0.5 (*dark red*); 0.5 ≤ *I*/*I*_0_ ≤ 0.8 (*light red*). *F*, structure of Ub (Protein Data Bank: 1D3Z), where residues that exhibited diminished *I*/*I*_0_ values in (*D*) are colored as follows: *I*/*I*_0_ < 0.5 (*dark red*); 0.5 ≤ *I*/*I*_0_ ≤ 0.8 (*light red*). *E* and *F*, residues not observed in these NMR experiments are indicated by an *asterisk*. The hydrophobic patch residues L8, I44, and V70 are labeled. PRE, paramagnetic relaxation enhancement; Ub, ubiquitin.

Rpn1^214–355^ naturally contains two cysteines, C246 and C252, which are located on opposite sides of the same helix (Fig. 4, *A* and *B*). Two single-cysteine Rpn1^214–355^ variants were produced: Rpn1^214–355(C246)^ and Rpn1^214–355(C252)^, wherein the specified cysteine remained present, whereas the other cysteine was mutated to serine. A nitroxide paramagnetic spin label ((1-oxyl-2,2,5,5-tetramethyl-3-pyrroline-3-methyl) methanesulfonate [MTSL]) was covalently attached through a disulfide bond to the remaining single cysteine in each Rpn1^214–355^ variant. This process enabled quantification of intermolecular distances through paramagnetic relaxation enhancement (PRE) effects induced by MTSL, whereby NMR signal intensities decreased for resonances corresponding to residues within ∼25 Å of MTSL (30). In other words, the NMR spectrum of an isotopically enriched protein would exhibit diminished signal intensities if binding to Rpn1^214–355^ occurs nearby the MTSL-attached helix, whereas no effect would be observed if the protein interacts with Rpn1^214–355^ at a location far from MTSL. Neither of these mutations nor the attachment of MTSL affected the functionality of Rpn1^214–355^; both MTSL-labeled variants were able to associate with polyUb and UBL domains, exhibiting negligible differences in NMR signal positions compared with equivalent binding experiments with nonmutated Rpn1^214–355^.

A ^1^H–^15^N NMR spectrum was recorded for a sample containing an equimolar amount of ^15^N-^d^K11-Ub_2_ and Rpn1^214–355(C246∼MTSL)^. Excess ascorbate was subsequently added to reduce the unpaired electron of MTSL, thereby quenching the paramagnetic effect of MTSL, after which another spectrum was recorded. Significant differences in NMR signal intensities were evident between the two spectra (Fig. 4*C*, *red*), indicating that the affected residues in ^15^N-^d^K11-Ub_2_ were within ∼25 Å of C246∼MTSL in Rpn1^214–355^. Notably, the majority of signal attenuations corresponded to residues in and around the hydrophobic patch of Ub.

This experiment was repeated with an equimolar mixture of ^15^N-^d^K11-Ub_2_ and Rpn1^214–355(C252∼MTSL)^. NMR signal attenuations were present in the hydrophobic patch region (Fig. 4*C*, *blue*), although they were substantially weaker in this case, thereby indicating that the distal Ub in K11-linked Ub_2_ binds Rpn1^214–355^ nearer to C246 than to C252. Because C246 points toward the solvent-exposed “front” side of Rpn1, whereas C252 points toward the “back” side (Fig. 4*B*), these results suggest that K11-linked Ub_2_ binds across the solvent-exposed surface of Rpn1 and in close proximity to C246. This is physically cogent, as the rear of Rpn1 is obstructed by the ATPase ring in the proteasome assembly, thus rendering any potential binding surface there inaccessible.

To determine if chain directionality (*i.e.*, if the distal and proximal domains in Ub_2_ are differentiated during binding) is a factor in the association of Rpn1^214–355^ and K11-linked Ub_2_, these PRE experiments were also performed using K11-linked Ub_2_ with the proximal Ub ^15^N-enriched (^15^N-^p^K11-Ub_2_). Intriguingly, a similar effect was observed for ^15^N-^p^K11-Ub_2_: significant NMR signal attenuations were exhibited in the presence of equimolar Rpn1^214–355(C246∼MTSL)^, whereas weaker attenuations were produced with equimolar Rpn1^214–355(C252∼MTSL)^ (Fig. 4*D*). This result corroborates the conclusion that K11-linked Ub_2_ binds nearby C246 and across the solvent-exposed surface of Rpn1^214–355^. The strikingly similar PRE profiles for the distal Ub and proximal Ub (Fig. 4, *C* and *D*) indicate that K11-linked Ub_2_ does not exhibit directionality when interacting with Rpn1^214–355^; thus, Rpn1^214–355^ does not appear to distinguish between the two Ubs in K11-linked Ub_2_. Notably, each Ub domain consistently displayed attenuations in signals corresponding to the hydrophobic patch region (Fig. 4, *E* and *F*)—an indication that these PRE effects actually probed the binding event.

**Figure 5:**
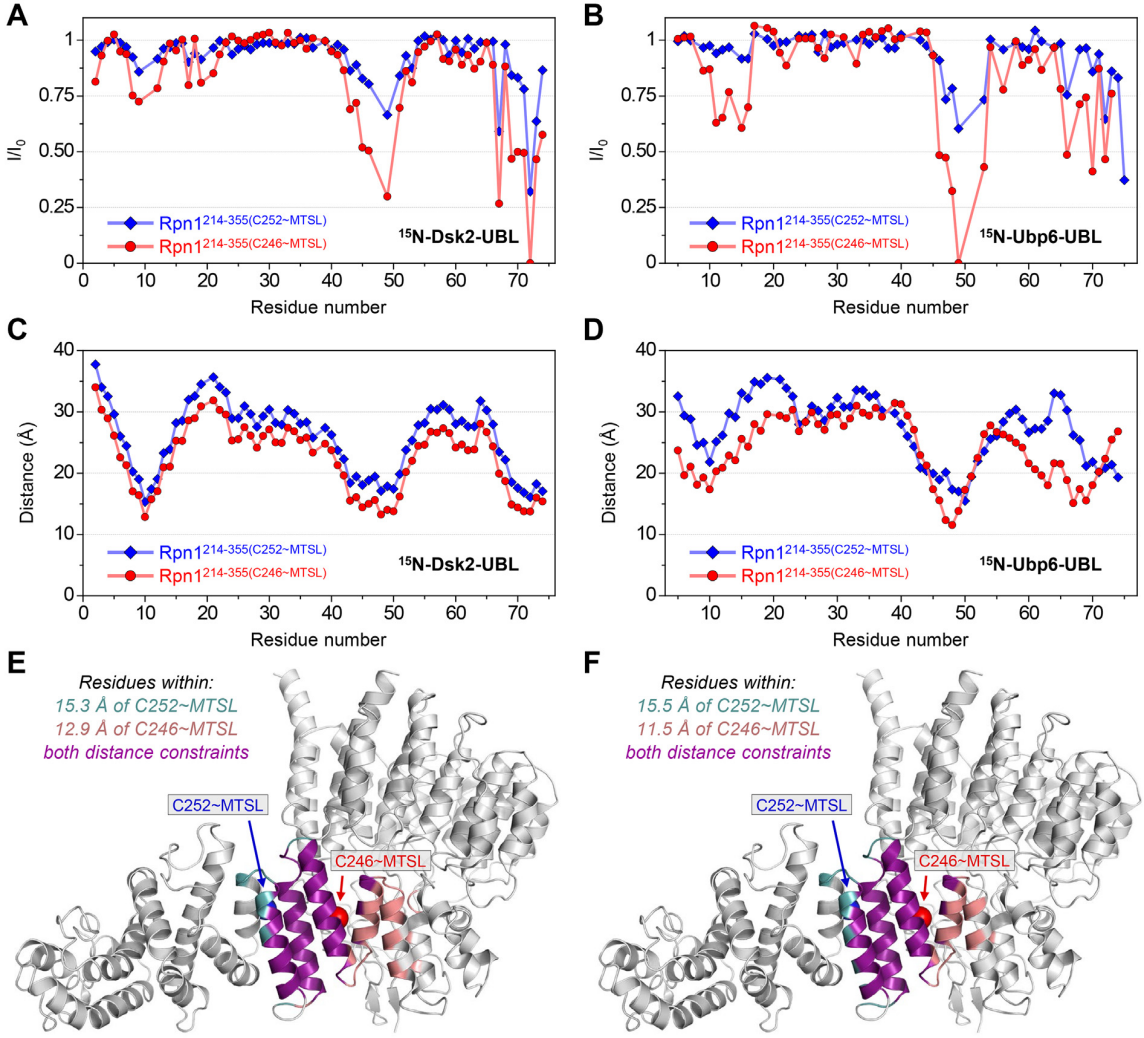
**Pinpointing the binding site in Rpn1**^**214–355**^**for Dsk2-UBL and Ubp6-UBL.***A* and *B*, PREs (*I*/*I*_0_) in (*A*) ^15^N-Dsk2-UBL or (*B*) ^15^N-Ubp6-UBL when mixed with an equimolar amount (150 μM of each protein) of Rpn1^214–355(C246~MTSL)^ (*red circles*) or Rpn1^214–355(C252~MTSL)^ (*blue diamonds*). *C* and *D*, the distance between backbone amides in (*C*) ^15^N-Dsk2-UBL or (*D*) ^15^N-Ubp6-UBL and the unpaired electron of MTSLs in Rpn1^214–355(C246~MTSL)^ (*red circles*) or Rpn1^214–355(C252~MTSL)^ (*blue diamonds*), as calculated by SLfit (50). *E* and *F*, mapping residues on the structure of Rpn1 (Protein Data Bank: 5MPC), where Rpn1 residues that may constitute the UBL-binding region are colored. *E*, residues within 12.9 Å of C246~MTSL are *pink*, residues within 15.3 Å of C252~MTSL are *teal*, and residues within both distance constraints are *purple*; these values correspond to the minimum interaction distances for Dsk2-UBL seen in (*C*). *F*, residues within 11.5 Å of C246~MTSL are *pink*, residues within 15.5 Å of C252~MTSL are *teal*, and residues within both distance constraints are *purple*; these values correspond to the minimum interaction distances for Ubp6–UBL seen in (*D*). *E* and *F*, the location of MTSL attached to C246 is indicated by a *red sphere*, whereas the location of MTSL attached to C252 is indicated by a *blue sphere*. MTSL, (1-oxyl-2,2,5,5-tetramethyl-3-pyrroline-3-methyl) methanesulfonate; PRE, paramagnetic relaxation enhancement; UBL, Ub-like.

PRE experiments were also performed with the UBL domains of Dsk2 and Ubp6. Significant residue-specific differences in NMR signal intensities were observed in the spectra of ^15^N-Dsk2-UBL and ^15^N-Ubp6-UBL when mixed with equimolar Rpn1^214–355(C246∼MTSL)^ or Rpn1^214–355(C252∼MTSL)^ (Fig. 5, *A* and *B*). As seen for K11-linked Ub_2_, signal attenuations were more severe with Rpn1^214–355(C246∼MTSL)^, indicating that C246 is closer than C252 to the UBL-binding surface in Rpn1^214–355^.

To further pinpoint the binding site location in Rpn1^214–355^, intermolecular distances between each MTSL and corresponding residues in ^15^N-Dsk2-UBL were quantified from the observed PREs using the in-house Matlab program SLfit (30). This analysis showed that the UBL domain of Dsk2 binds Rpn1^214–355^ as close as ∼13 Å from C246∼MTSL and ∼15 Å from C252∼MTSL (Figs. 5*C* and S7, *A*–*C*); this information was used to identify Rpn1^214–355^ residues that could be in contact with Dsk2-UBL. Rpn1^214–355^ residues within a ∼13 Å radius from C246∼MTSL and residues within a ∼15 Å radius from C252∼MTSL were mapped onto the structural model of Rpn1 (Protein Data Bank [PDB]: 5MPC) (Fig. 5*E*). Residues that satisfied these distance constraints constitute the likely binding site for Dsk2-UBL.This dual-distance determination suggested that the novel recognition site in Rpn1^214–355^ may consist of several adjacent helices, spanning from residue ∼220 to ∼300. Notably, the T1 site in Rpn1^391–642^ is also composed of multiple adjacent helices spread out over ∼90 residues (6). Qualitatively, this analysis indicated that the Dsk2-UBL:Rpn1^214–355^ recognition surface is in relative proximity (within ∼15 Å) of residues 246 and 252 in Rpn1^214–355^, as opposed to the residues further downstream (residues ∼320–355).

Likewise, we determined that Ubp6-UBL interacts with Rpn1^214–355^ at a minimum distance of ∼11.5 Å from C246∼MTSL and ∼15.5 Å from C252∼MTSL (Figs. 5*D* and S7, *D*–*F*). These distances were remarkably similar to those for Dsk2-UBL, and the recognition site was mapped to the same helical region in both cases (Fig. 5, *E* and *F*); therefore, we concluded that Rpn1^214–355^ recognizes Dsk2-UBL and Ubp6-UBL through analogous binding modes. Although comparable results were obtained from PRE experiments with K11-linked Ub_2_ (Fig. S8), those distances should be interpreted cautiously because of the apparent lack of directionality in K11-linked Ub_2_ binding of Rpn1^214–355^. In this case, PREs may reflect positional averaging across all bound states, and because multiple arrangements of K11-linked Ub_2_ are likely sampled during binding of Rpn1^214–355^, the respective PRE values may not accurately correspond to intermolecular distances. Nevertheless, the similar PRE profiles among all moieties suggest that K11-linked Ub_2_ binds to the same site on Rpn1^214–355^ as Dsk2-UBL and Ubp6-UBL. The location of this Ub/UBL-recognition site is likely in the region encompassing residues ∼220 to ∼300 of Rpn1, although this approximation is dependent upon the accuracy of structural models of Rpn1. Note that these PRE effects also provide additional confirmation of close contacts between Rpn1^214–355^ and the proteasomal signals studied here.

### MS–MS analysis of crosslinked products identifies recognition site in Rpn^214–355^

In a parallel attempt to narrow down the location of the novel Ub/UBL-recognition site in Rpn1, the aforementioned crosslinking reactions with Rpn1^214–355^ and Ub^T9Bpa^ (shown in Fig. 2*A*) were digested with trypsin and subjected to LC–MS to identify photocrosslinking sites in the Ub^T9Bpa^–Rpn1^214–355^ complex. We identified four different peptides of Rpn1^214–355^ that were crosslinked to the ^7^TL[*Bpa*]GK^11^ fragment of Ub^T9Bpa^, primarily covering the region from residue 288 to residue 318 in Rpn1 (Table S2). These data indicate that the novel Ub/UBL-recognition site is likely situated in the proximity of residues 288 to 318 in Rpn1 and also serve as an additional verification method of the interaction between Rpn1^214–355^ and Ub.

**Figure 6:**
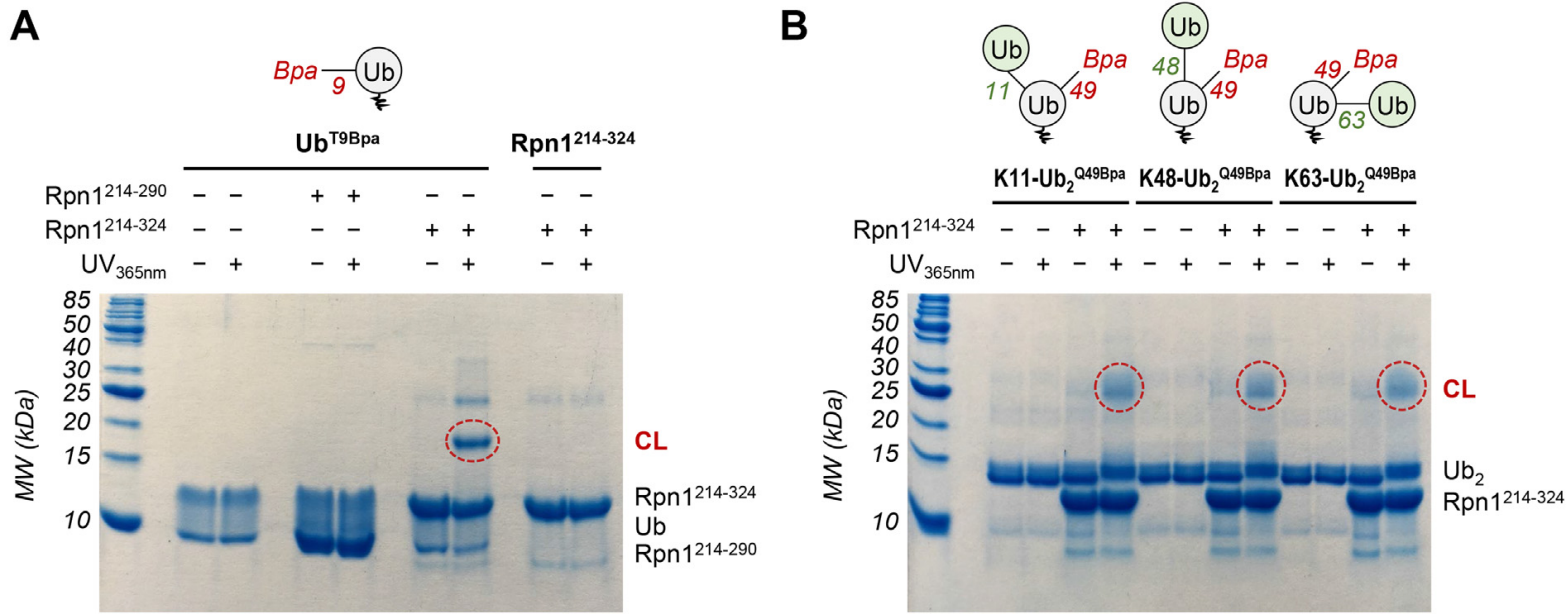
**Ub and Ub**_**2**_**crosslink with Rpn1**^**214–324**^**but not Rpn1**^**214–290**^**.** SDS-PAGE gels showing the results of exposure to UV_365nm_ irradiation of (*A*) Bpa-containing Ub and Rpn1^214–290^ or Rpn1^214–324^; (*B*) Bpa-containing Ub_2_ moieties and Rpn1^214–324^. *A* and *B*, the moiety written in *bold* above the gel is always present in the respective gel lanes, whereas addition of Rpn1^214–290^, Rpn1^214–324^, or exposure to UV_365nm_ irradiation is indicated by *plus/minus* symbols. Coomassie staining was performed. Crosslinked products (CL) are indicated by *red circles*. A schematic of each Ub or Ub_2_ moiety is shown at the *top*, wherein Bpa is *red*, the Bpa-attached Ub is *gray*, the distal Ub (if present) is *green*, and the residue number of the linked lysine (if present) is *green*. Bpa, *p*-benzoyl-l-phenylalanine; Ub, ubiquitin.

Encouraged by these results, we truncated Rpn1^214–355^ even further and generated two shorter Rpn1 constructs: Rpn1^214–290^ and Rpn1^214–324^. These truncation sites were carefully positioned in the flexible regions between helices so as to not disrupt the global structure of Rpn1. Additional crosslinking reactions with Bpa-containing Ub were performed to determine if these truncated Rpn1 constructs retain the ability to recognize Ub. Intriguingly, a prominent band corresponding to a crosslinked product was evident in the reaction with Ub^T9Bpa^ and Rpn1^214–324^, whereas no crosslinking was detected in the reaction with Ub^T9Bpa^ and Rpn1^214–290^ (Fig. 6*A*). These results support our initial observation that Ub crosslinks with residues 288 to 318 in Rpn1, as removal of these residues abolishes crosslinking. Rpn1^214–324^ also crosslinked with K11-Ub_2_^Q49Bpa^, K48-Ub_2_^Q49Bpa^, and K63-Ub_2_^Q49Bpa^ in a similar manner as Rpn1^214–355^ (Fig. 6*B*).

**Figure 7:**
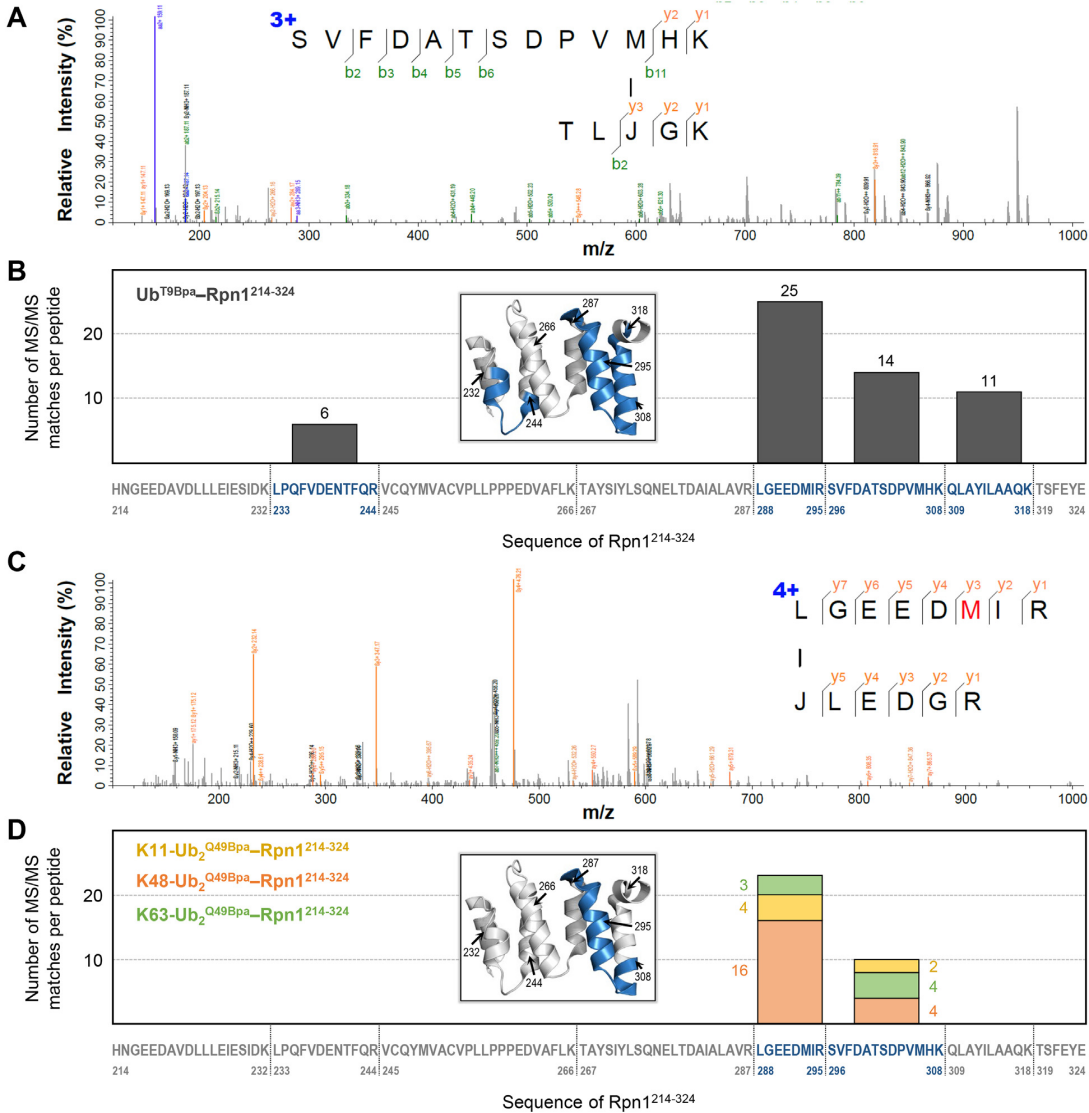
**MS–MS analysis of Ub**^**T9Bpa**^**–Rpn1**^**214–324**^**and Ub**_**2**_^**Q49Bpa**^**–Rpn1**^**214–324**^**crosslinked complexes.***A*, representative MS–MS spectrum of one of the Ub^T9Bpa^–Rpn1^214–324^ crosslinked peptides. *B*, the number of MS–MS matches per Rpn1^214–324^ peptide are plotted for Ub^T9Bpa^–Rpn1^214–324^. *C*, representative MS–MS spectrum of one of the Ub_2_^Q49Bpa^–Rpn1^214–324^ crosslinked peptides. *D*, the number of MS–MS matches per Rpn1^214–324^ peptide are plotted for K11-Ub_2_^Q49Bpa^–Rpn1^214–324^ (*yellow*), K48-Ub_2_^Q49Bpa^–Rpn1^214–324^ (*orange*), and K63-Ub_2_^Q49Bpa^–Rpn1^214–324^ (*green*). *A* and *C*, the sequence of the crosslinked peptides is shown, wherein Bpa is represented by *J*. *B* and *D*, *vertical dotted lines* indicate trypsin digestion sites in the sequence of Rpn1^214–324^, with residue numbers shown. Matched peptides are *blue*, whereas unmatched peptides are *gray*. The found crosslinked peptides are mapped on the structure of Rpn1 (Protein Data Bank: 4CR2) in the inset using the same coloring scheme; the trypsin digestion sites are indicated. Bpa, *p*-benzoyl-l-phenylalanine; Ub, ubiquitin.

Next, we performed in-gel digestion of the proteins from gel bands corresponding to crosslinked Ub^T9Bpa^–Rpn1^214–324^ or Ub_2_^Q49Bpa^–Rpn1^214–324^ (*circled* in Fig. 6) and subjected the samples to LC–MS as before (Fig. 7). This in-gel digestion of Ub^T9Bpa^–Rpn1^214–324^ confidently identified the same three peptides in Rpn1^214–324^ crosslinked to Ub^T9Bpa^, corresponding to residues 288 to 318 in Rpn1 (Table S3). One additional crosslinked peptide was observed for the Ub^T9Bpa^–Rpn1^214–324^ complex (Table S3), corresponding to residues 233 to 244 in Rpn1. This indicates that the novel recognition site in Rpn1 may span across multiple helices (Fig. 7*B*), which is perhaps unsurprising given our PRE results and the fact that the T1 site in Rpn1^391–642^ is also composed of multiple helices (6). Meanwhile, all three Ub_2_^Q49Bpa^–Rpn1^214–324^ digests identified crosslinking between the ^49^[*Bpa*]LEDGR^54^ fragment of Ub_2_^Q49Bpa^ and residues 288 to 308 in Rpn1 (Fig. 7, *C* and *D* and Tables S4–S6). The Ub_2_^Q49Bpa^–Rpn1^214–324^ samples were less concentrated than the Ub^T9Bpa^–Rpn1^214–324^ sample, which may explain why fewer peptide matches were observed for the dimers. Nevertheless, these MS results consistently demonstrate crosslinking between monoUb or Ub_2_ species and residues 288 to 308 in Rpn1, thereby implicating these residues in signal recognition.

To support our crosslinking findings, we performed NMR binding experiments to examine the recognition properties of the truncated Rpn1 constructs. Rpn1^214–290^ did not elicit any noticeable perturbations in the NMR signals of ^15^N-^d^K11-Ub_2_ (Fig. S9*A*), even at a twofold molar excess. Meanwhile, Rpn1^214–324^ produced substantial shifts and attenuations in the NMR signals of ^15^N-^d^K11-Ub_2_ (Fig. S9*B*), and the visual pattern of these perturbations (Fig. 3*E*) was comparable to that of the equivalent experiment with Rpn1^214–355^ and ^15^N-^d^K11-Ub_2_ (Fig. 3*B*). The *K*_*d*_ for the binding between ^15^N-^d^K11-Ub_2_ and Rpn1^214–324^ was measured as 42 ± 21 μM (Fig. 3*E*), essentially identical to the corresponding value of 44 ± 16 μM for Rpn1^214–355^ (Fig. 3*B*).

These NMR experiments indicate that the novel Ub/UBL-recognition site in Rpn1 is entirely situated within residues 214 to 324, as removing residues 325 to 355 did not alter the binding properties of the Rpn1 construct. Meanwhile, the full complement of this recognition site is not contained within residues 214 to 290 in Rpn1. These results agree with MS–MS analysis of crosslinked samples, which show that an essential portion of the Ub/UBL-recognition site in Rpn1 consists of residues 288 to 318.

Taken together, our results identify a novel site in Rpn1^214–355^ that recognizes Ub, polyUb, and UBL signals with physiologically relevant affinities and exhibits binding preferences that are remarkably similar to those of the T1 site in Rpn1^391–642^. To differentiate between these recognition sites in Rpn1, we name the site in Rpn1^214–355^ the NT site (*N*-terminal to *T*oroid), as it is N-terminal to the toroidal PC repeat region. Intriguingly, K11-linked Ub_2_, K48-linked Ub_2_, Dsk2-UBL, Ubp6-UBL, and Rad23-UBL all appear to interact with the NT site in Rpn1^214–355^, supporting previous observations that binding sites in Rpn1 are shared among various polyUb species and UBL domains (24).

## Discussion

In this study, we discovered a previously unidentified recognition site for polyUb and UBL signals in the Rpn1 region encompassing residues 214 to 355. Even though it is a small fragment of a larger protein, isolated Rpn1^214–355^ is folded and predominantly helical—as anticipated based on structural models of full-length Rpn1. This region of Rpn1 does not contain any of the classical helix–turn–helix PC repeats that are purportedly involved in recognizing proteasomal signals (6, 15); however, taking our CD data and the existing structural models of Rpn1 into account, Rpn1^214–355^ likely contains several unclassified helix–turn–helix motifs. Although these helix–turn–helix motifs exhibit insufficient sequence homology to be considered members of the PC repeat family, they interact with polyUb and UBL domains nonetheless.

Photocrosslinking and NMR experiments unequivocally demonstrated that Rpn1^214–355^ associates with Ub, Ub_2_, and multiple UBL domains. Therefore, Rpn1 contains at least two recognition sites for proteasomal signals—the novel NT site identified here and the T1 site identified previously (6, 15). Intriguingly, both sites appear to exhibit similar binding affinity hierarchies: *Rad23-UBL >> Dsk2-UBL ≈ K11-linked Ub*_*2*_ *> Ubp6-UBL > K48-linked Ub*_*2*_ *> Ub*; meanwhile, neither site interacts with the UBL domain of purported shuttle protein Ddi1 (24). Although Rpn1^214–355^ displayed slightly higher *K*_*d*_ values than those for Rpn1^391–642^, the majority of interactions involving Rpn1^214–355^ occurred with physiologically relevant affinity. Interestingly, the NT site in Rpn1^214–355^ seems to be shared among polyUb and UBL domains; this promiscuity was also observed for Rpn1^391–642^ and full-length Rpn1 (24). Thus, Rpn1 appears to contain multiple shared signal recognition sites with analogous binding preferences, rather than one distinct recognition site for each signal.

Full-length Rpn1 has previously been shown to strongly bind UBL domains, with *K*_*d*_ values of ∼12 μM for Dsk2-UBL and ∼2 μM for Ubp6-UBL (11). Yet, respective affinities for Rpn1^391–642^ (*K*_*d*_: ∼22 μM, ∼40 μM) and Rpn1^214–355^ (*K*_*d*_: ∼48 μM, ∼104 μM) are weaker (7). This discrepancy may be explained by the existence of multiple recognition sites nearby each other in Rpn1. After a UBL domain binds and subsequently dissociates from a binding site in full-length Rpn1, the UBL domain may quickly reassociate with a nearby site in Rpn1; this increased local concentration effect results in enhanced apparent affinity. However, isolated Rpn1^214–355^ and Rpn1^391–642^ constructs do not contain the full complement of binding sites. Thus, reassociation of Dsk2-UBL or Ubp6-UBL becomes less likely, thereby diminishing the measured binding affinity.

The NT site location in Rpn1 was ultimately narrowed down to a region of ∼110 residues spanning across multiple helix–turn–helix motifs, just as for the T1 site (6). Paramagnetic spin-labeling experiments suggested that the NT site is likely contained within the region of residues 220 to 300, whereas MS–MS analysis of the trypsinized crosslinked products detected crosslinking across residues 233 to 244 and 288 to 318 in Rpn1. It is important to note that this MS–MS analysis only probed the spatial proximity between Rpn1^214–324^ and two residues in Ub, whereas many Ub residues are involved in the association with Rpn1^214–324^ based on NMR CSP data. On the other hand, PRE experiments probed the distance to Rpn1 for nearly every residue in Ub, thereby sampling a larger recognition surface. Notably, both Bpa and MTSL are dynamic moieties whose flexibility should be considered when interpreting these results. Although we cannot conclude that the novel Ub/UBL-recognition site is solely contained within residues 288 to 318 of Rpn1, it is clear that this region is an essential component of the recognition site, as crosslinking and NMR experiments with Rpn1^214–290^ demonstrated that removal of this region abolishes binding. Collectively, our experiments showed that the entirety of the NT site is located within Rpn1^214–324^.

**Figure 8:**
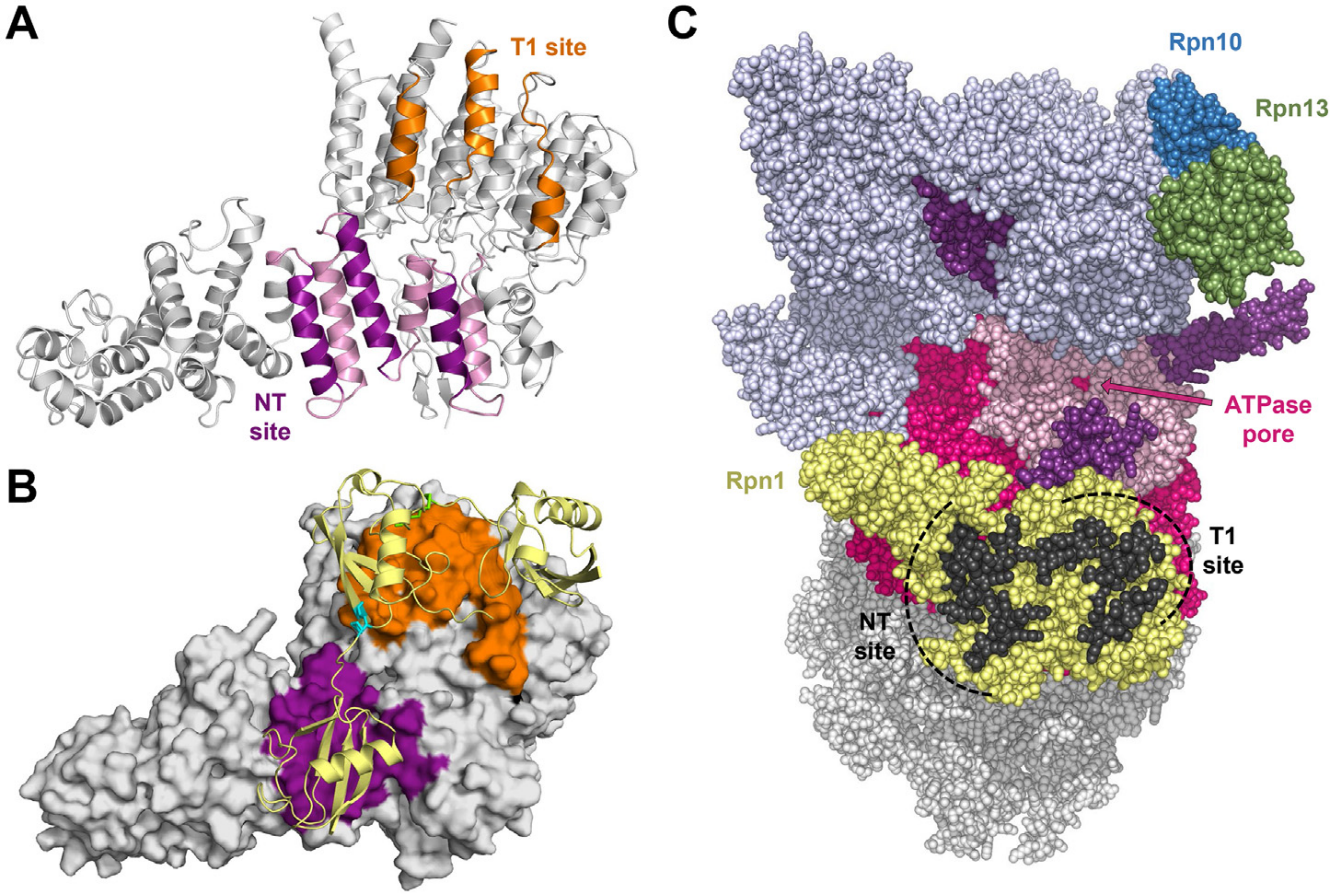
**Rpn1 contains two adjacent Ub/UBL-binding sites that may promote bidentate signal recognition.***A*, structure of Rpn1 (Protein Data Bank [PDB]: 5MPC); the three helices comprising the T1 site are *orange* (6), the three forward-facing solvent-exposed helices contained within the NT site (residues 214–324) are *purple*, and the remaining residues within the NT site are *pink*. In this orientation, the back side of Rpn1 (contacting the ATPase ring) is behind the page, whereas the solvent-exposed front side of Rpn1 is sticking out of the page. *B*, surface representation of Rpn1 (PDB: 5MPC), with the same coloring as in (*A*), where a HADDOCK-generated model of polyUb is bound across both binding sites in Rpn1 simultaneously. This Ub_3_ (*yellow*) model consists of one K48 linkage (*green*) and one K11 linkage (*cyan*). *C*, structure of the 26S proteasome (PDB: 4CR2), showing the RP (*colored*) and one half of the CP (*gray*). Rpn1 is *yellow*, with the solvent-exposed helices of both putative binding site regions colored *black*; helices corresponding to the T1 site and NT site are indicated. Rpn10 is *blue* and Rpn13 is *green*. The ATPases are colored as follows: the AAA+ domains are *fuchsia*, the coiled–coil domains are *purple*, and the OB ring is *light pink*. The ATPase pore is indicated by the cavity in the center of the OB ring. The remaining RP subunits are periwinkle. CP, core particle; NT, *N*-terminal to *T*oroid; RP, regulatory particle; Ub, ubiquitin; UBL, Ub-like.

Site-directed spin-labeling experiments also suggested that Ub/UBL binding occurs along the solvent-exposed “front” surface of Rpn1. Three solvent-exposed helices are located within residues 214 to 324 of Rpn1 (Fig. 8*A*); two of these helices correspond to the crosslinked peptides identified by MS–MS analysis, spanning across residues 233 to 244 and 288 to 308 in Rpn1 (Fig. 7). Notably, the T1 site in Rpn1 also consists of three solvent-exposed helices (6) (Fig. 8*A*). Despite the NT and T1 sites being sequentially separated by over 200 residues, their spatial proximity is glaringly apparent (Fig. 8*A*): at their closest point, only ∼7 Å separates the helical backbones of these two regions. Remarkably, both sites recognize signals with comparable affinity profiles and are positioned within close spatial proximity to each other on the solvent-exposed surface of Rpn1. Thus, we speculate that these sites offer a platform for multidentate binding, whereby polyUb or polyUb·UBL can simultaneously anchor itself to multiple sites on Rpn1.

The feasibility of multivalent recognition was examined computationally using HADDOCK (31), whereby polyUb was docked across both binding sites in Rpn1 (Fig. 8*B*). The structure of K48-linked Ub_2_ bound to the T1 site in Rpn1 was used as the initial model (6), and HADDOCK was utilized to extend Ub_2_ into a longer polyUb chain concurrently bound to the NT site (see *Experimental procedures* section). However, extension through a single K48-linked Ub did not allow for simultaneous K48-linked Ub_3_ binding across both sites in Rpn1 (Fig. S10*A*). Instead, K48-linked Ub_4_—often considered to be the minimal efficient proteasomal signal (32)—was required to bridge the gap between the two sites (Fig. S10*B*). Interestingly, the optimal Ub_3_ docking arrangement involved extension of K48-linked Ub_2_ through a branched K11-linked Ub, a byproduct of K11's ideal positioning near the NT site in Rpn1 (Fig. 8*B*), thereby forming branched K11/K48-linked Ub_3_. This observation is in line with previous studies, which have shown that branched K11/K48-linked polyUb is an enhanced degradation signal for Rpn1, especially when compared with polyUb linked through only K48 (28, 33, 34). Nevertheless, we would like to emphasize that these models are not intended to be interpreted stringently but rather serve as a visual demonstration that simultaneous polyUb recognition across both sites in Rpn1 is physically feasible.

There are many advantages of the proteasome containing multiple sites to anchor a polyUb chain rather than a single site. First, it may increase the probability of an initial binding event occurring. Furthermore, it may decrease the likelihood of the signal prematurely dissociating from the proteasome or being disassembled by deubiquitinases before the substrate is fed into the CP. Multisite binding may also optimize positioning of the substrate closer to the translocation point, rather than dangling freely on the end of a flexible and dynamic chain.

Mapping which Rpn1 residues constitute both binding sites shows that a considerable portion of the solvent-exposed surface of Rpn1 is involved in signal recognition (Fig. 8*C*). Besides these two sites, additional sites for Ddi1-UBL and Ubp6-UBL have also been proposed (6, 14)—although their validity is debated (11, 24)—thereby cluttering the binding landscape of Rpn1 even further. Perhaps this region would be best described not as a discrete number of individual recognition sites but rather as one elongated recognition surface that can accommodate an extensive assortment of signals with diverse lengths and topologies. Indeed, it was recently shown that Rpn1 is particularly important for the degradation of substrates tagged with multiple monoUbs, multiple polyUb chains, and shuttle proteins (23). Notably, all these moieties consist of numerous signals often connected through flexible linker regions; it is possible that the broad binding platform of Rpn1 is adept at accommodating substrates with scattered signals, whereas the smaller receptors (Rpn10 and Rpn13) are unable to do so. As mentioned before, specific branched polyUb chains, which in some cases may exhibit signaling and/or structural properties comparable to multipolyubiquitination (34, 35), enhance substrate degradation through Rpn1 (28, 33, 34). Thus, Rpn1 may act as the proteasomal equivalent of a “bottom-feeder”—able to recognize signals that are less compatible with the other receptors.

The elongated binding surface of Rpn1 may accommodate diversity with respect to the length of substrates and their corresponding signal(s), which might explain why the efficiency of Rpn1 improves as substrates and/or their associated polyUb chains increase in size, particularly when substrates are also associated with shuttle proteins (23). The distance between the ATPase pore—where the unfolded substrate is ultimately fed into the CP—and the binding surface on Rpn1 varies from ∼100 to ∼170 Å (Fig. 8*C*). This broad distance range may provide enough space for polyubiquitinated conjugates with atypically large substrates and/or polyUb chains to associate along the farther edge of the binding surface on Rpn1 and remain in proximity to the ATPase pore, whereas shorter polyUb signals attached to smaller substrates bind Rpn1 nearer to the ATPase pore. Conversely, the lesser distances from the ATPase pore to the sites in Rpn10 (∼90 Å) and Rpn13 (∼105 Å), as well as the narrower distribution of the respective binding surfaces, may prevent the recognition of larger complexes by these two receptors.

In summary, this work identified and characterized a novel binding site for Ub, polyUb, and UBL moieties in an unexpected region of Rpn1. This NT site exhibits similar signal recognition preferences to the T1 site in Rpn1. Because of the comparable nature and proximity of these two sites in Rpn1, we speculated that they may support multivalent binding, thereby improving the efficiency of signal recognition, substrate translocation, and substrate degradation. Furthermore, the elongated binding surface of Rpn1 may be responsible for processing polyubiquitinated substrates “decorated” with shuttle proteins that populate a large conformational space, whereas Rpn10 and Rpn13 may be more adept at recognizing smaller complexes. These findings offer new mechanistic insights into signal recognition processes that are at the core of the UPS.

## Experimental procedures

### Protein expression and purification

Rpn1^214–355^ from *Saccharomyces cerevisiae* was expressed as a His_6_-Smt3-Rpn1^214–355^ fusion construct in *Escherichia coli* BL21(DE3) Codon Plus cells; a similar process was described previously (7). One liter cultures of Luria broth media supplemented with 50 μg/ml kanamycin and 100 μg/ml chloramphenicol were grown at 37 °C until the absorbance at 600 nm reached ∼0.6, after which IPTG was added to a final concentration of 1 mM. Cells were incubated at 37 °C for an additional 3 to 4 h and then harvested by centrifugation; from this point onward, all steps were performed at 4 °C or on ice. Cells were resuspended in 30 ml of 50 mM Tris, 300 mM potassium chloride, at pH 8.0. DNAse I (Worthington Biochemical Corp) was added to a final concentration of 10 μg/ml, and one EDTA-free protease inhibitor tablet (Thermo Scientific) was dissolved in the solution. Cells were lyzed by sonication, and the lysate was clarified by ultracentrifugation. The resulting pellet was resuspended in 25 ml of wash buffer (50 mM Tris, 500 mM potassium chloride, 750 mM urea, pH 8.0) and 1% Triton X-100. The solution was briefly sonicated, rocked for 30 min, and reclarified by ultracentrifugation. The pellet was again resuspended in 25 ml of wash buffer, briefly sonicated, rocked for 30 min, and reclarified by ultracentrifugation. Finally, the pellet was resuspended in 40 ml of extraction buffer (50 mM Tris, 500 mM potassium chloride, 20 mM imidazole, 7 M urea, 3 mM tris(2-carboxyethyl)phosphine (TCEP), pH 8.0). As before, DNAse I and one protease inhibitor tablet were added to the solution, which was then rocked overnight.

The resulting solution was clarified by ultracentrifugation and filtered. The supernatant was then loaded onto a 5 ml HisTrap (GE Healthcare) column pre-equilibrated with extraction buffer. A shallow gradient with 50 mM Tris, 500 mM potassium chloride, 20 mM imidazole, 3 mM TCEP, at pH 8.0 was used to slowly remove urea from the buffer over several hours, thereby allowing the protein to refold on the column. His_6_-Smt3-Rpn1^214–355^ was eluted from the column with 50 mM Tris, 500 mM potassium chloride, 250 mM imidazole, 3 mM TCEP, at pH 8.0. His_6_-ULP1 was added to the solution to cleave His_6_-Smt3 from Rpn1^214–355^, and the solution was dialyzed overnight against 50 mM Hepes, 500 mM potassium chloride, 3 mM TCEP, 5% glycerol, at pH 7.6. The solution was then loaded onto a pre-equilibrated 5 ml HisTrap column, and Rpn1^214–355^ was collected in the flow-through. Gel filtration was used to separate monomeric Rpn1^214–355^ from any oligomeric species, whereby the solution was loaded onto a HiLoad 16/60 Superdex 75 pg (GE Healthcare) column equilibrated with 50 mM Hepes, 500 mM potassium chloride, 3 mM TCEP, at pH 7.6.

Ub monomers from *Homo sapiens* (36), Bpa-containing Ub monomers from *H. sapiens* (27), and the UBL domains from *S. cerevisiae* (Rad23-UBL, Dsk2-UBL, Ubp6-UBL, and Ddi1-UBL) (11, 12, 13, 37) were expressed in *E. coli* cells and purified as described previously. The UBL domains corresponded to the following residues: 1 to 73 for Rad23-UBL; 2 to 77 for Dsk2-UBL; 2 to 81 for Ubp6-UBL; and 1 to 80 for Ddi1-UBL. Uniprot accession numbers are as follows: P0CG48 (Ub); P32628 (Rad23); P48510 (Dsk2); P43593 (Ubp6); P40087 (Ddi1); and P38764 (Rpn1).

### PolyUb chain assembly

PolyUb chains were assembled *via* established controlled-length enzymatic protocols (30, 38), which enabled isotopic enrichment of specific domains (36). Conjugating enzymes Ube2S (39) and E2-25K (Ube2K) (36) were used to make K11 linkages and K48 linkages, respectively. Specific mutations controlled polyUb length and linkage architecture: K11R/K48R/K63R for the distal Ub and K63R/D77 for the proximal Ub. K11R, K48R, and D77 mutations prevented unwanted chain elongation, whereas K63R mutations blocked Ube2S from making a minor fraction of K63 linkages (39). Reactions were performed overnight at 37 °C in the presence of activating enzyme E1 and 2 mM ATP, after which Ub_2_ was separated from unreacted Ub by cation chromatography.

This protocol was also followed for making the Bpa-containing Ub_2_ moieties (27), which contained a proximal Ub with Bpa incorporated at position 49 and a C-terminal His_6_ tag instead of D77. Bpa-containing K63-linked Ub_2_ was assembled by utilizing the linkage-specific conjugating enzyme complex of Ubc13 and Mms2 (40). Because of the high linkage specificity of this complex, K63R Ub was used as the distal domain.

### CD spectroscopy

CD spectra were recorded in continuous mode on a Jasco J-810 spectropolarimeter equilibrated to 20 °C, with a sampling range of 320 to 190 nm and a scanning speed of 50 nm/min. Rpn1^214–355^ was prepared at a concentration of 0.2 mg/ml in 20 mM potassium phosphate, 50 mM sodium fluoride, 500 μM TCEP, at pH 7.4. Spectra for the buffer and for Rpn1^214–355^ were recorded in triplicate.

Ellipticity data across the three runs were averaged and buffer subtracted. The processed data were analyzed by the DICHROWEB server (41). Deconvolution was successful with the CONTINLL (42) and CDSSTR (43) methods in combination with reference sets 4, 7, SP175, and SMP180 (44, 45, 46).

### Bpa photocrosslinking

Photocrosslinking samples were prepared in 50 mM Hepes, 50 mM potassium chloride, 1 mM TCEP, at pH 7.6 and contained 50 μM of Rpn1^214–355^ (or Rpn1^214–324^ or Rpn1^214–290^) and 25 μM of the Bpa-containing Ub or Ub_2_ species. Samples were incubated on ice and exposed to UV irradiation at λ = 365 nm (UV_365nm_) for 1 h, as detailed elsewhere (27), after which they were resolved by SDS-PAGE and visualized by Coomassie staining and silver staining.

### MS–MS analysis of crosslinked products

Crosslinking products of Rpn1^214–355^ and Ub^T9Bpa^ were precipitated with acetone, and the ensuing pellet was resuspended in either 50 mM ammonium bicarbonate, 6 M urea or 50 mM ammonium bicarbonate, and 8 M guanidinium chloride (GuHCl). Cysteines were reduced with 5 mM DTT for 30 min and alkylated with 15 mM iodoacetamide for 30 min in the dark at room temperature. The samples were diluted with 50 mM ammonium bicarbonate to reduce the concentration of urea or GuHCl below 1 M, prior to digestion with sequencing-grade trypsin (Promega) at a 1:100 w/w enzyme:substrate ratio for 12 h at 37 °C. Samples were lyophilized, dissolved in 8 M GuHCl, and then subjected to a second digestion with trypsin (1:50 w/w). Crosslinking products of Rpn1^214–324^ and Ub^T9Bpa^ or Ub_2_^Q49Bpa^ were prepared in a similar manner following in-gel digestion protocols described previously (47).

The resulting peptide mixtures were desalted using C18 Stage-tips and subjected to LC–MS analysis using a Q Exactive Plus Orbitrap mass spectrometer coupled to nano-HPLC. The peptides were resolved by reversed-phase chromatography on 0.075 × 180 mm fused silica capillaries (Agilent J&W) packed with Reprosil reversed-phase material (Dr Maisch GmbH). The peptides were eluted with a linear 60-min gradient of 5 to 28% acetonitrile with 0.1% formic acid, followed by a 15-min gradient of 28 to 95% acetonitrile with 0.1% formic acid, and a 10-min wash of 95% acetonitrile with 0.1% formic acid (at flow rates of 0.15 μl/min). MS was performed in positive mode using an *m/z* range of 300 to 1800, a resolution of 60,000 for MS1, and a resolution of 15,000 for MS2; repetitively full MS scans were followed by high-energy collisional dissociation of the ten most dominant ions selected from the first MS scan.

Identification of crosslinked peptides was performed following analysis of the MS RAW files by pLink (version 2.3.9, *via* pFind Studio (48)), using Bpa as the crosslinker and trypsin as the digestion enzyme, with a maximum of three missed cleavage sites. Carbamidomethylation of cysteines was set as a fixed modification, and oxidation of methionines was set as a variable modification. Peptide N-terminal and lysine carbamylation was included as a variable modification for the in-solution digestion of Ub^T9Bpa^–Rpn1^214–355^ in urea. Considered peptide mass was set to 400 to 10,000 kDa and peptide length was set to 4 to 40 amino acid residues. Precursor tolerance was set to 10 ppm, whereas fragment tolerance was set to 20 ppm. Results were filtered by application of a precursor mass accuracy of ±10 ppm and 5% false discovery rate.

Searches were conducted against a database containing the sequences of Ub and Rpn1^214–355^ supplemented with the sequences of 293 known potential contaminant proteins (total of 295 sequences). The search results were also validated (data not shown) against a larger database composed of these 295 sequences supplemented with *E. coli* protein sequences (Uniprot version 2021_01) that contained 4686 sequences in total (two target proteins, 4393 *E. coli* proteins, and 293 known potential contaminant proteins).

### NMR spectroscopy

NMR experiments were performed at 25 °C on Bruker Avance III 600 MHz and 800 MHz spectrometers equipped with cryoprobes. NMR samples were prepared in 50 mM Hepes, 50 mM potassium chloride, 1 mM TCEP, 0.02% NaN_3_, 5 to 10% D_2_O, at pH 7.6. Initial protein concentrations ranged from 50 to 150 μM. Binding experiments were performed by adding stepwise volumes of a concentrated ligand and recording a ^1^H-^15^N SOFAST-HMQC spectrum at each point. NMR data were processed with TopSpin 3.5 (Bruker) and analyzed with Sparky (University of California) (49).

CSPs (Δδ) were calculated for each residue, as follows:

(1) $$\Delta \delta =\sqrt{{\left(\Delta \delta_{H}\right)}^{2}+{\left(\Delta \delta_{N}/5\right)}^{2}}$$

where Δδ_*H*_ and Δδ_*N*_ correspond to chemical shift differences for the ^1^H and ^15^N resonances, respectively.

The *K*_*d*_ was determined by fitting experimental CSPs for respective titration points to a single-site binding model using the in-house Matlab program Kdfit (40), as follows:

(2) $$\Delta \delta =\Delta \delta_{\max}\frac{\left[P_{t}\right]+\left[L_{t}\right]+K_{d}-\sqrt{{\left(\left[P_{t}\right]+\left[L_{t}\right]+K_{d}\right)}^{2}-4\left[P_{t}\right]\left[L_{t}\right]}}{2\left[P_{t}\right]}$$

where [*P*_*t*_] and [*L*_*t*_] are the total molar concentrations of protein and ligand at each titration point; Δδ_max_ is the CSP value at saturation; *K*_*d*_ was treated as a global fitting parameter.

Site-directed spin labeling was performed by covalently attaching a paramagnetic nitroxide spin label, MTSL, to a single cysteine residue in Rpn1^214–355(C246)^ and Rpn1^214–355(C252)^ (30). The ^15^N-enriched protein of interest was then mixed with an equimolar amount of Rpn1^214–355(C246∼MTSL)^ or Rpn1^214–355(C252∼MTSL)^. A ^1^H–^15^N heteronuclear single quantum coherence spectrum of the mixture was recorded in the paramagnetic (oxidized) state of MTSL. Excess ascorbate was then added to the sample, after which another ^1^H–^15^N heteronuclear single quantum coherence spectrum was recorded with MTSL in the diamagnetic (reduced) state.

PRE effects were determined by quantifying the signal intensity ratio (*I*/*I*_0_) between the oxidized (*I*) and reduced (*I*_0_) states (30). The location of the unpaired electron of MTSL and its distance to each residue were determined from the experimental intensity ratios using the in-house Matlab program SLfit (50, 51). PyMol was utilized to identify and visualize residues that satisfied respective distance constraints.

### Structural modeling with HADDOCK

The HADDOCK2.2 Web server (31) was utilized to produce a model of polyUb docked across both binding sites in Rpn1 simultaneously. The initial coordinates file was generated by aligning a structure of full-length Rpn1 (PDB: 5MPC) with a structure of K48-linked Ub_2_ bound to the T1 site in Rpn1^412–625^ (PDB: 2N3W). This Rpn1•K48-linked Ub_2_ model was docked with a single Ub (PDB: 1D3Z) or another K48-linked Ub_2_ (PDB: 2N3W), thereby creating a Ub_3_ or Ub_4_ moiety. Active residues for Rpn1 were defined as residues with >40% solvent accessibility that also satisfied both sets of distance restraints from PRE experiments with Dsk2-UBL and Ubp6-UBL (L225, E226, S229, I230, K266, S270, and S274). Active residues for Ub were defined as residues that form the hydrophobic patch (L8, I44, and V70). Passive residues were automatically defined as residues within 6.5 Å of active residues. Unambiguous restraints were used to preserve the existing K48 linkage in Ub_2_ as well as introduce a new K11 linkage or K48 linkage (28, 52, 53). The flexibility of Rpn1 was defined automatically. Ub_2_ and Ub residues composing the isopeptide linkages (10–12, 47–49, and 70–76) were considered semiflexible, whereas residues of a free C terminus (70–76) were considered fully flexible.

Docking was performed following standard HADDOCK procedures. Energy minimization generated 2000 rigid-body docking structures; the 200 best structures according to ambiguous interaction restraint energy were subjected to semiflexible refinement. The resulting structures were refined in water and clustered with a fraction of common contacts cutoff of 0.6. The models shown (Figs. 8*B* and S10) are the highest scoring structures from the highest scoring cluster.

## Data availability

The mass spectrometry proteomics data were deposited to the ProteomeXchange Consortium *via* the PRIDE (54) partner repository under the dataset identifier PXD027128. Further data are available upon reasonable request from David Fushman: fushman@umd.edu.

## Supporting information

This article contains supporting information (6, 41, 42, 43, 44, 45, 46, 48, 50, 55, 56, 57, 58, 59).

## Conflict of interest

The authors declare that they have no conflicts of interest with the contents of this article.
