# Supplementary material for: A novel recognition site for polyubiquitin and ubiquitin-like signals in an unexpected region of proteasomal subunit Rpn1
Source: J Biol Chem. 2021 Aug 6;297(3):101052. doi: 10.1016/j.jbc.2021.101052 (PMC8405992; doi:10.1016/j.jbc.2021.101052)
Supplement: Figures S1–S10 and Tables S1–S6 [file mmc1.pdf]

Supporting Information for:

A novel recognition site for polyubiquitin and ubiquitin-like signals in an unexpected region of proteasomal subunit Rpn1

Andrew J. Boughton[1], Leonard Liu[1], Tali Lavy[2], Oded Kleifeld[2], and David Fushman[1,*]

[1] Department of Chemistry and Biochemistry, Center for Biomolecular Structure and Organization, University of Maryland, College Park, MD 20742, USA

[2] Faculty of Biology, Technion-Israel Institute of Technology, Technion City, Haifa 3200003, Israel

*Correspondence: David Fushman (fushman@umd.edu)

This file includes:

Figures S1 to S10

Tables S1 to S6

Supporting Figures

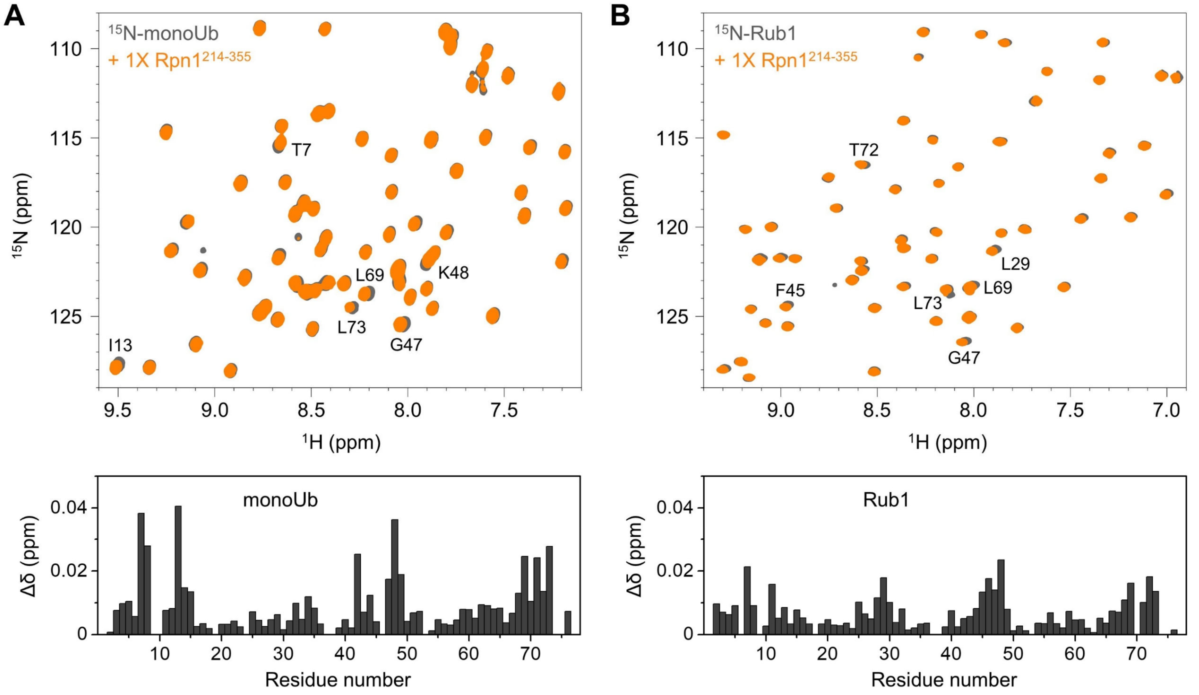

Figure S1: MonoUb and Rub1 weakly interact with Rpn1$^{214-355}$. Overlaid ^{1}H-^{15}N NMR spectra of: (A, top) 100 μM ^{15}N-monoUb (grey), 100 μM ^{15}N-monoUb plus equimolar Rpn1$^{214-355}$ (orange); (B, top) 150 μM ^{15}N-Rub1 (grey), 150 μM ^{15}N-Rub1 plus equimolar Rpn1$^{214-355}$ (orange). Select residues are indicated. Residue-specific CSPs (Δδ) for: (A, bottom) 100 μM ^{15}N-monoUb plus equimolar Rpn1$^{214-355}$; (B, bottom) 150 μM ^{15}N-Rub1 plus equimolar Rpn1$^{214-355}$.

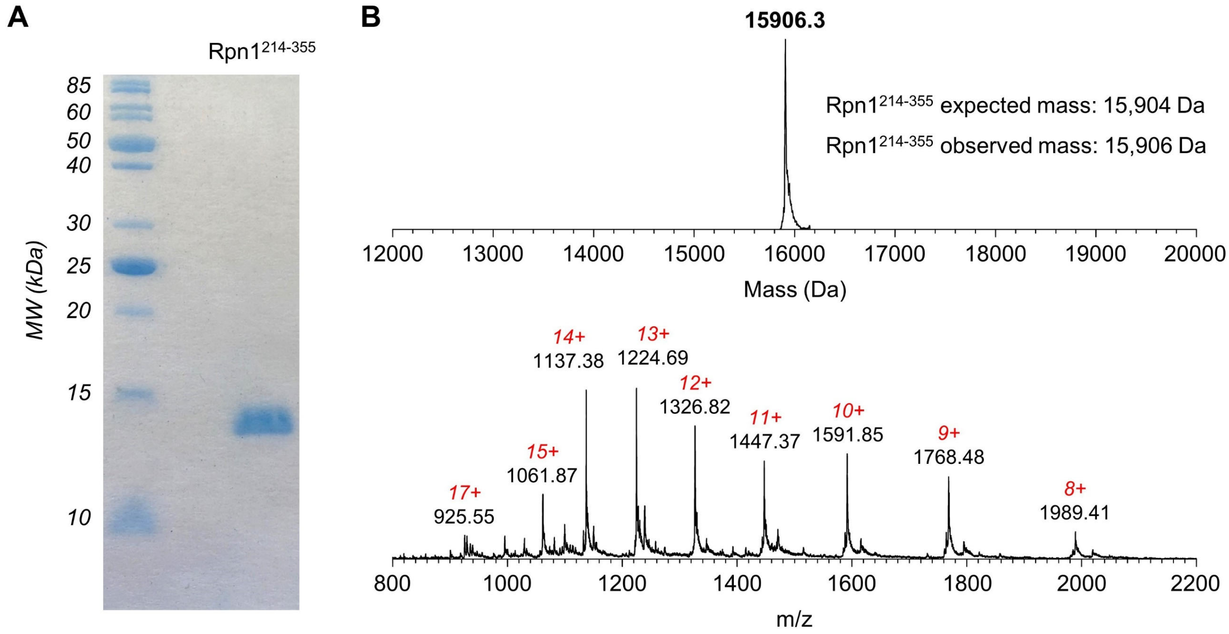

Figure S2: Preliminary characterization of Rpn1$^{214\text{-}355}$. (A) SDS-PAGE gel of Rpn1$^{214\text{-}355}$, visualized by Coomassie staining. Rpn1$^{214\text{-}355}$ migrated close to its expected mass of ~16 kDa. (B) ESI-MS spectrum of Rpn1$^{214\text{-}355}$, showing good agreement between expected and observed masses.

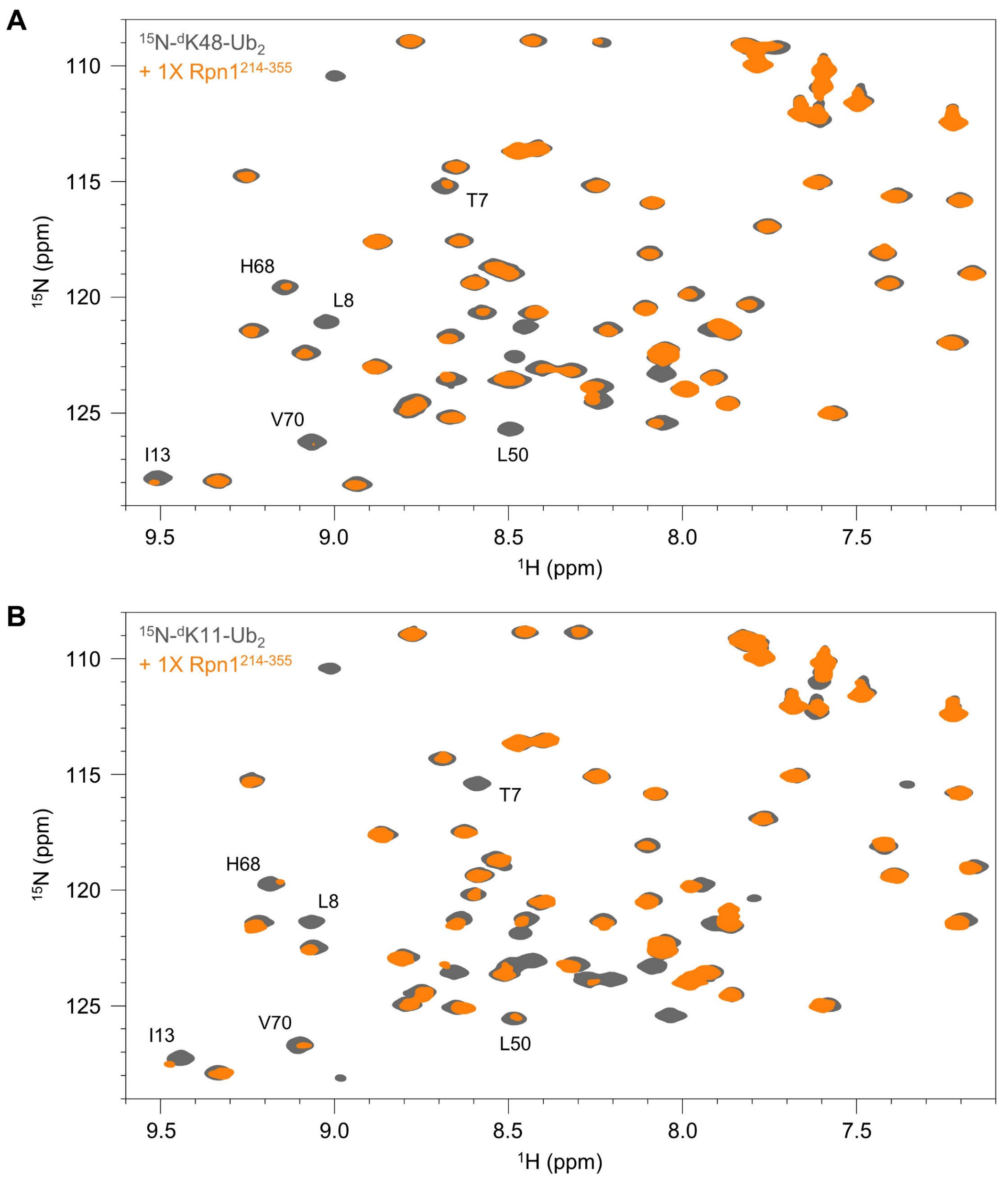

Figure S3: Rpn1$^{214-355}$ binds K48-linked Ub$_2$ and K11-linked Ub$_2$. Overlaid ^{1}H-^{15}N NMR spectra of: (A) 150 μM ^{15}N-^{d}K48-Ub$_2$ (grey), 150 μM ^{15}N-^{d}K48-Ub$_2$ plus equimolar Rpn1$^{214-355}$ (orange); (B) 150 μM ^{15}N-^{d}K11-Ub$_2$ (grey), 150 μM ^{15}N-^{d}K11-Ub$_2$ plus equimolar Rpn1$^{214-355}$ (orange). Select residues are indicated.

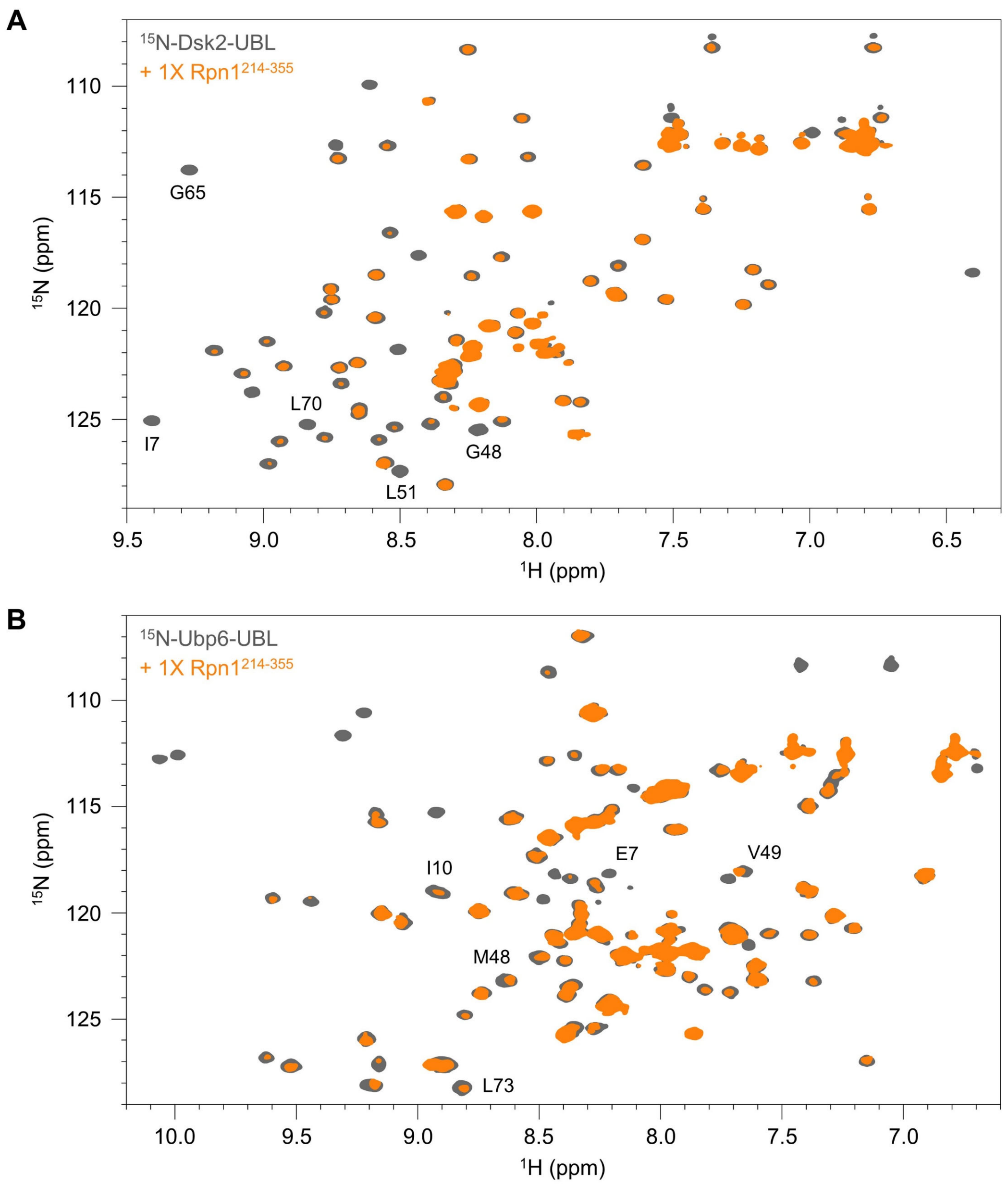

Figure S4: Rpn1$^{214-355}$ binds the UBL domains of Dsk2 and Ubp6. Overlaid ^{1}H-^{15}N NMR spectra of: (A) 150 µM ^{15}N-Dsk2-UBL (grey), 150 µM ^{15}N-Dsk2-UBL plus equimolar Rpn1$^{214-355}$ (orange); (B) 150 µM ^{15}N-Ubp6-UBL (grey), 150 µM ^{15}N-Ubp6-UBL plus equimolar Rpn1$^{214-355}$ (orange). Select residues are indicated.

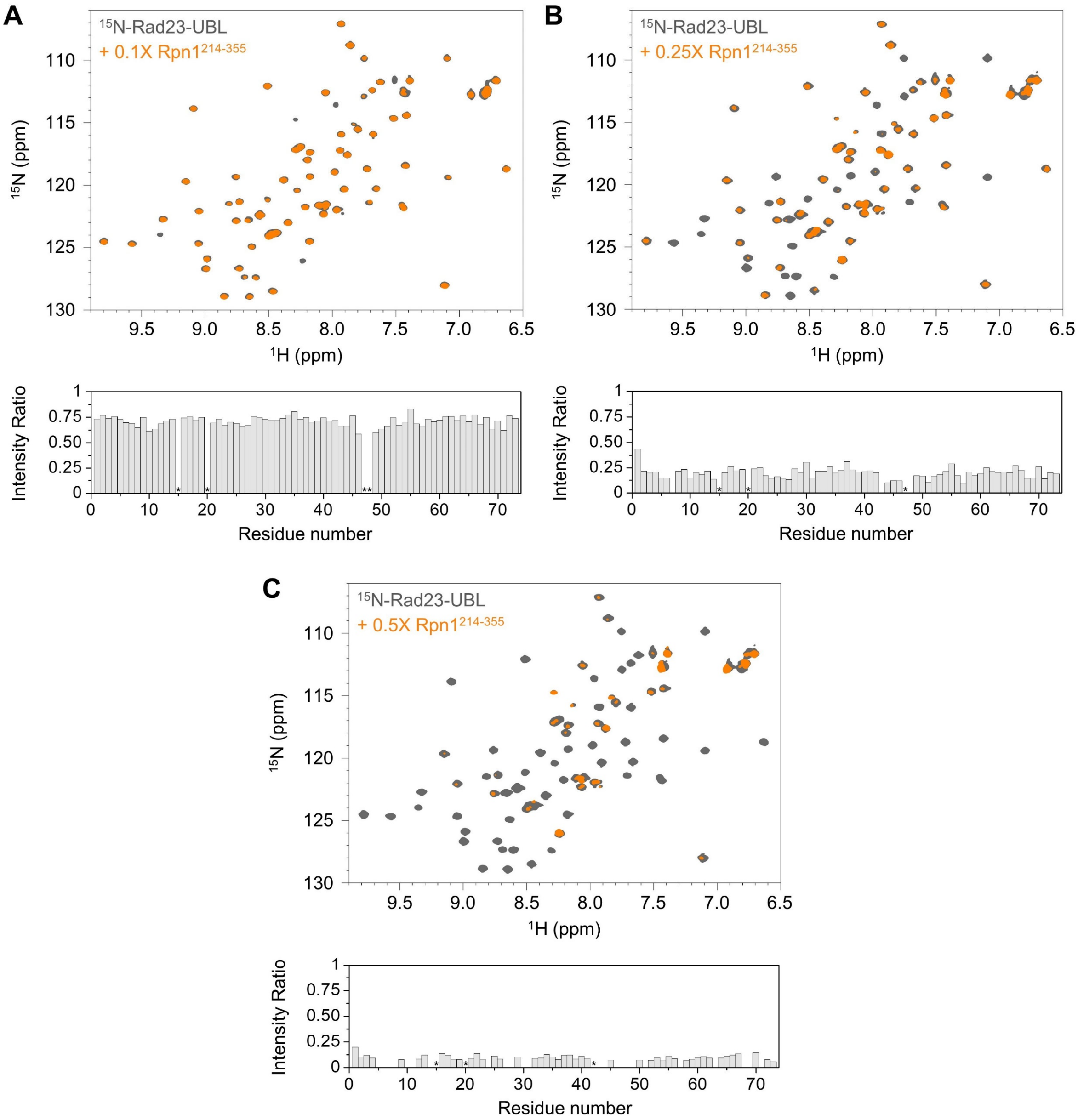

Figure S5: The UBL domain of Rad23 exhibits NMR signal attenuations upon Rpn1$^{214\text{-}355}$ addition. Overlaid ^{1}H-^{15}N NMR spectra of: (A, top) 100 μM ^{15}N-Rad23-UBL (grey), 100 μM ^{15}N-Rad23-UBL plus 0.1X Rpn1$^{214\text{-}355}$ (orange); (B, top) 100 μM ^{15}N-Rad23-UBL (grey), 100 μM ^{15}N-Rad23-UBL plus 0.25X Rpn1$^{214\text{-}355}$ (orange); (C, top) 100 μM ^{15}N-Rad23-UBL (grey), 100 μM ^{15}N-Rad23-UBL plus 0.5X Rpn1$^{214\text{-}355}$ (orange). Quantification of NMR signal intensity ratios on a per-residue basis for: (A, bottom) 100 μM ^{15}N-Rad23-UBL vs. 100 μM ^{15}N-Rad23-UBL plus 0.1X Rpn1$^{214\text{-}355}$; (B, bottom) 100 μM ^{15}N-Rad23-UBL vs. 100 μM ^{15}N-Rad23-UBL plus 0.25X Rpn1$^{214\text{-}355}$; (C, bottom) 100 μM ^{15}N-Rad23-UBL vs. 100 μM ^{15}N-Rad23-UBL plus 0.5X Rpn1$^{214\text{-}355}$. An intensity ratio of one indicates no signal disappearance, while an intensity ratio of zero indicates total signal disappearance. Residues denoted with an asterisk were not observed in the NMR spectra and were not included in analysis.

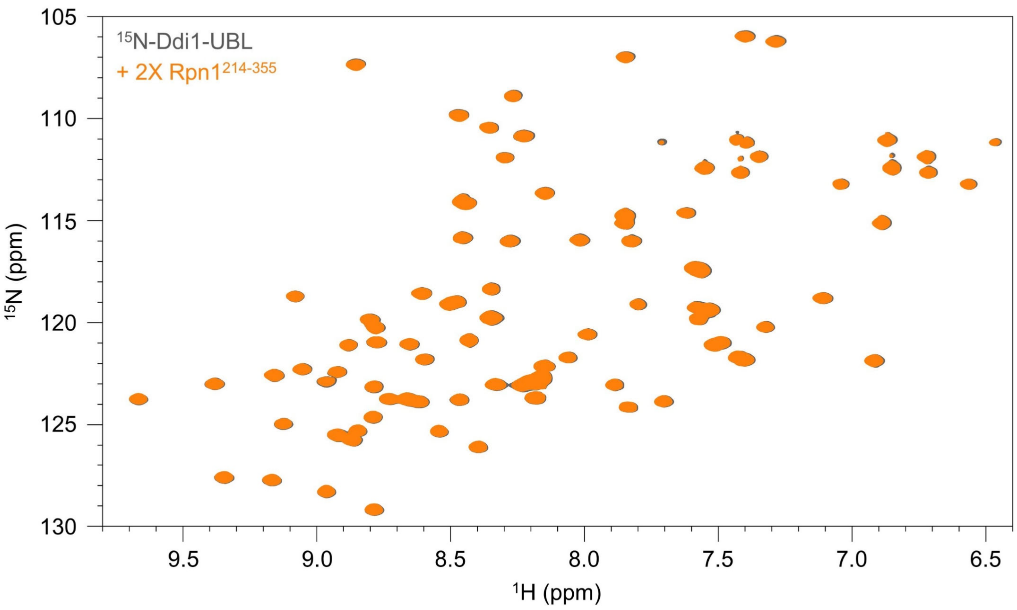

Figure S6: Rpn1$^{214\text{-}355}$ does not bind the UBL domain of Ddi1. Overlaid ^{1}H-^{15}N NMR spectra of: 100 µM ^{15}N-Ddi1-UBL (grey), 100 µM ^{15}N-Ddi1-UBL plus 2X Rpn1$^{214\text{-}355}$ (orange).

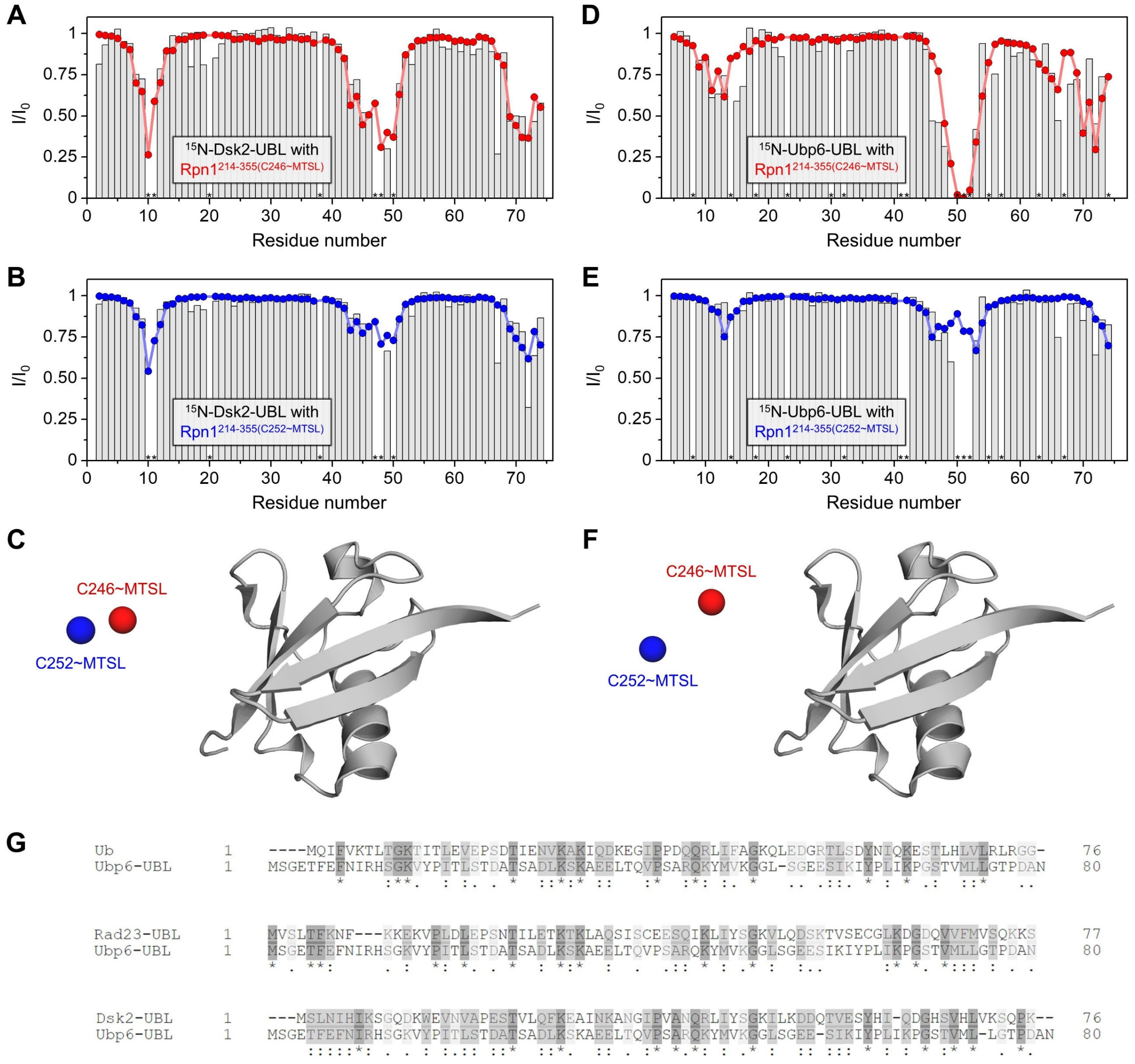

Figure S7: Analysis of PRE data for Dsk2-UBL and Ubp6-UBL. (A-B) PREs (I/I_0) for (A) ^{15}N-Dsk2-UBL with equimolar Rpn1$^{214\text{-}355(C246\sim MTSL)}$ and (B) ^{15}N-Dsk2-UBL with equimolar Rpn1$^{214\text{-}355(C252\sim MTSL)}$. Grey bars depict experimental PREs, while colored circles represent PREs back-calculated from the reconstructed MTSL position. Residues denoted with an asterisk were not observed in the NMR spectra and not included in the analysis. (C) Structure of Dsk2-UBL (PDB: 2BWF), where the reconstructed position of MTSL's unpaired electron is shown as a red (Rpn1$^{214\text{-}355(C246\sim MTSL)}$) sphere and blue (Rpn1$^{214\text{-}355(C252\sim MTSL)}$) sphere. (D-E) PREs for (D) ^{15}N-Ubp6-UBL with equimolar Rpn1$^{214\text{-}355(C246\sim MTSL)}$ and (E) ^{15}N-Ubp6-UBL with equimolar Rpn1$^{214\text{-}355(C252\sim MTSL)}$. Grey bars depict experimental PREs, while colored circles represent PREs back-calculated from the reconstructed MTSL position. Residues denoted with an asterisk were not observed in the NMR spectra and not included in the analysis. (F) Because the structure of Ubp6-UBL is unknown, analysis was performed with the structure of Dsk2-UBL (PDB: 2BWF), where the reconstructed position of MTSL's unpaired electron is shown as a red (Rpn1$^{214\text{-}355(C246\sim MTSL)}$) sphere and blue (Rpn1$^{214\text{-}355(C252\sim MTSL)}$) sphere. (G) Sequence alignments of Ubp6-UBL with Ub, Rad23-UBL, and Dsk2-UBL. The sequence of Ub is from *H. sapiens* and the sequences of the UBL domains are from *S. cerevisiae*.

Sequence identity is shown by asterisks (dark grey shading), while sequence similarity is indicated by a colon (medium grey shading) for strong similarity and a period (light grey shading) for weak similarity. Ubp6-UBL is substantially more similar to Dsk2-UBL (45.1% similarity) than to either Rad23-UBL (35.0% similarity) or Ub (35.8% similarity); consequently, the structure of Dsk2-UBL was chosen to represent the structure of Ubp6-UBL in (F). Alignment was performed using the Clustal Omega program (1).

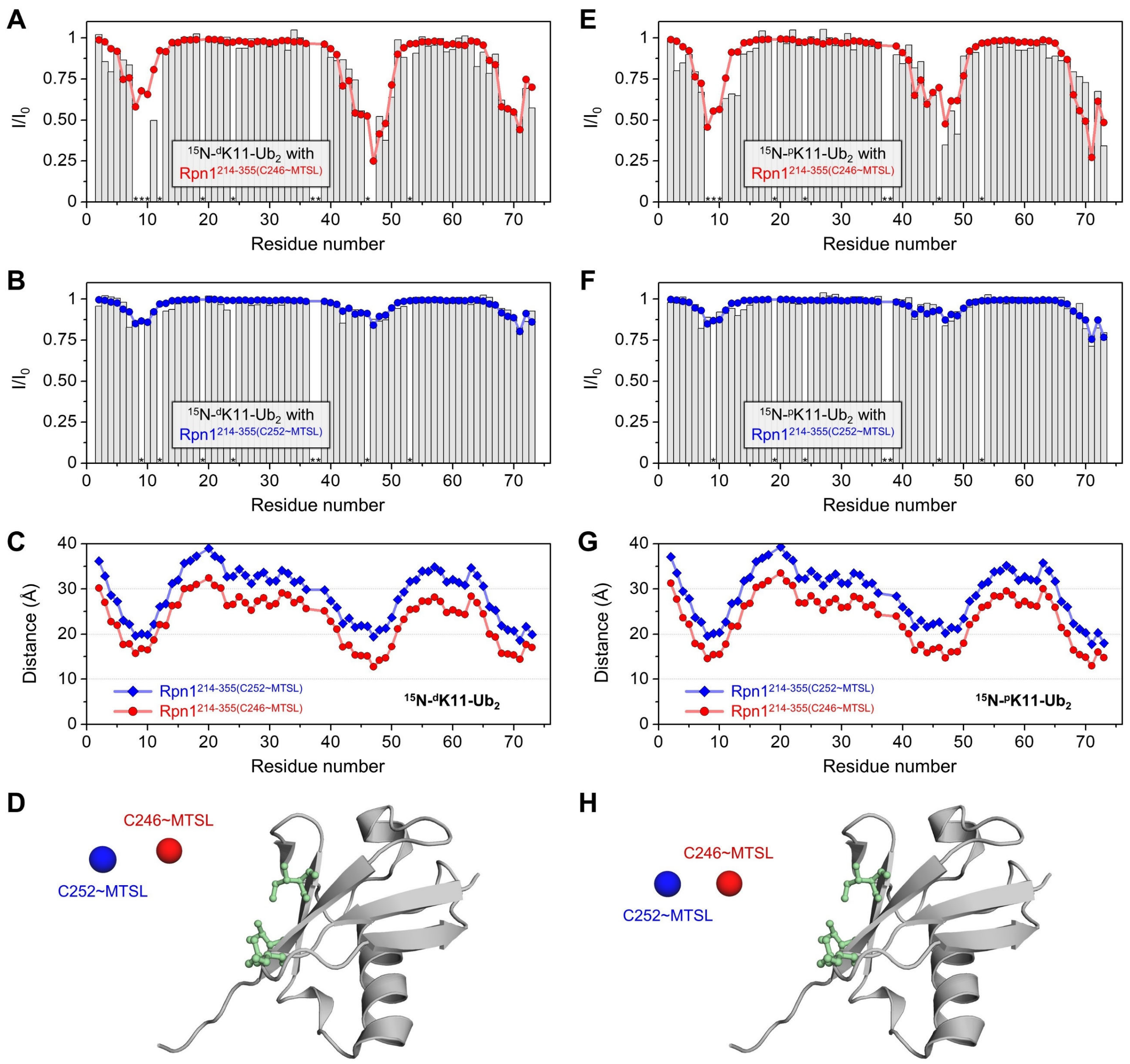

Figure S8: Analysis of PRE data for the distal and proximal domains of K11-linked Ub$_2$.
(A-B) PREs (I/I$_0$) for (A) ^{15}N-^{d}K11-Ub$_2$ with equimolar Rpn1$^{214\text{-}355(C246\sim MTSL)}$ and (B) ^{15}N-^{d}K11-Ub$_2$ with equimolar Rpn1$^{214\text{-}355(C252\sim MTSL)}$. Grey bars depict experimental PREs, while colored circles represent PREs back-calculated from the reconstructed MTSL position. Residues denoted with an asterisk were not observed in the NMR spectra and not included in the analysis. (C) The distance between backbone amides in ^{15}N-^{d}K11-Ub$_2$ and MTSL's unpaired electron in either Rpn1$^{214\text{-}355(C246\sim MTSL)}$ (red circles) or Rpn1$^{214\text{-}355(C252\sim MTSL)}$ (blue diamonds), as calculated by the in-house Matlab program SLfit (2).
(D) Structure of Ub (PDB: 1D3Z, representing the distal Ub in ^{15}N-^{d}K11-Ub$_2$), where the hydrophobic patch residues (L8, I44, and V70) are shown as green sticks and the reconstructed position of MTSL's unpaired electron is shown as a red (Rpn1$^{214\text{-}355(C246\sim MTSL)}$) sphere and blue (Rpn1$^{214\text{-}355(C252\sim MTSL)}$) sphere.
(E-F) PREs for (E) ^{15}N-^{p}K11-Ub$_2$ with equimolar Rpn1$^{214\text{-}355(C246\sim MTSL)}$ and (F) ^{15}N-^{p}K11-Ub$_2$ with equimolar Rpn1$^{214\text{-}355(C252\sim MTSL)}$. Grey bars depict experimental PREs, while colored circles represent PREs back-calculated from the reconstructed MTSL position. Residues denoted with an asterisk were not observed in the NMR spectra and not included in the analysis. (G) The distance between backbone amides in ^{15}N-

^{p}K11-Ub_2 and MTSL's unpaired electron in either Rpn1$^{214\text{-}355(C246\sim MTSL)}$ (red circles) or Rpn1$^{214\text{-}355(C252\sim MTSL)}$ (blue diamonds), as calculated by the in-house Matlab program SLfit (2). (H) Structure of Ub (PDB: 1D3Z, representing the proximal Ub in ^{15}N-^{p}K11-Ub_2), where the hydrophobic patch residues (L8, I44, and V70) are shown as green sticks and the reconstructed position of MTSL's unpaired electron is shown as a red (Rpn1$^{214\text{-}355(C246\sim MTSL)}$) sphere and blue (Rpn1$^{214\text{-}355(C252\sim MTSL)}$) sphere.

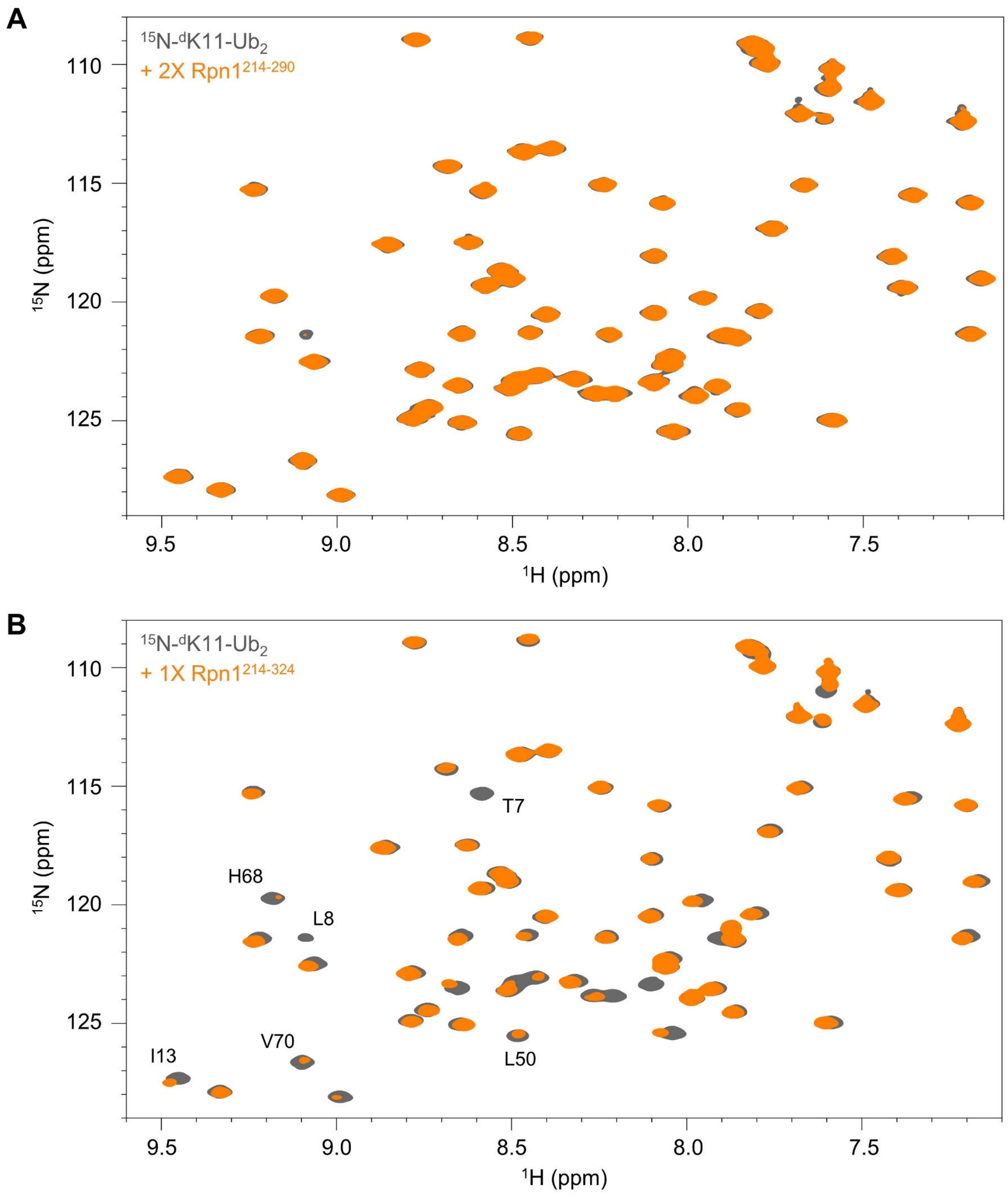

Figure S9: K11-linked Ub_2 binds $Rpn1^{214\text{-}324}$, but not $Rpn1^{214\text{-}290}$. Overlaid ^{1}H-^{15}N NMR spectra of: (A) 150 μM ^{15}N-$^{d}K11$-Ub_2 (grey), 150 μM ^{15}N-$^{d}K11$-Ub_2 plus 2X $Rpn1^{214\text{-}290}$ (orange); (B) 150 μM ^{15}N-$^{d}K11$-Ub_2 (grey), 150 μM ^{15}N-$^{d}K11$-Ub_2 plus equimolar $Rpn1^{214\text{-}324}$ (orange). Select residues are indicated.

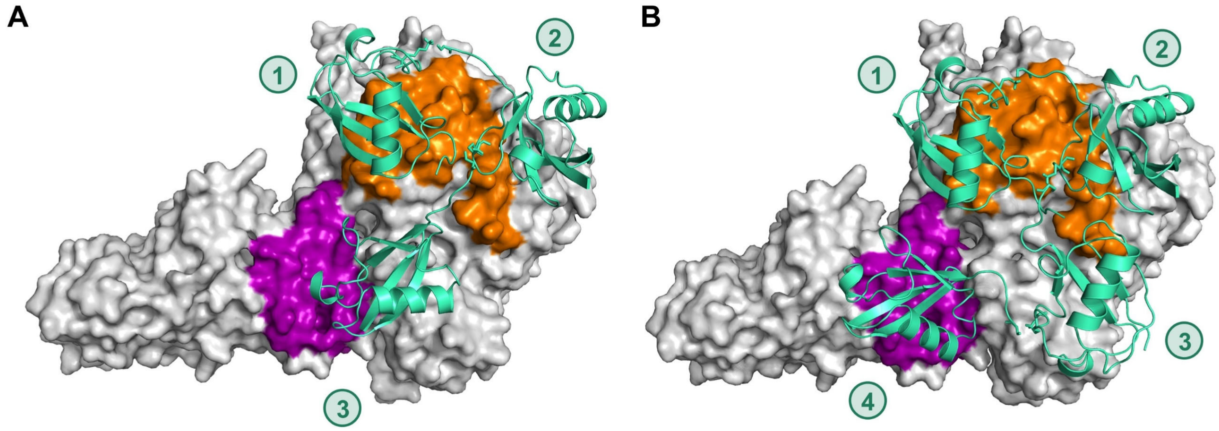

Figure S10: Modeling the multivalent binding of K48-linked polyUb across Rpn1. (A-B) Surface representation of Rpn1 (PDB: 5MPC); the three helices comprising the T1 site in Rpn1 are orange (3), while the three forward-facing solvent-exposed helices contained within the NT site (residues 214-324) are purple. In this orientation, the back side of Rpn1 is behind the page, while the front side of Rpn1 is sticking out of the page. A HADDOCK-generated model of (A) K48-linked Ub_3 (green) or (B) K48-linked Ub_4 (green) bound across both binding sites in Rpn1 simultaneously. Ub units in each polyUb are sequentially numbered, with the proximal Ub as number one and continuing to the distal Ub as number three (K48-linked Ub_3) or number four (K48-linked Ub_4). See Fig. 8B for an analogous model with branched K11/K48-linked Ub_3.

Supporting Tables

Table S1: Secondary structure characterization of Rpn1$^{214\text{-}355}$.

Secondary Structure Characterization of Rpn1$^{214\text{-}355}$ from PDB: 5MPC [a,b]				
	**Helices (per 100 residues)**	**Average helix length (residues)**	**Strands (per 100 residues)**	**Average strand length (residues)**
**STRIDE** [c]	6.3	11.3	0	0
**DSSP** [d]	6.3	10.7	0	0
Secondary Structure Prediction of Rpn1$^{214\text{-}355}$ from Experimental CD Data [e]				
	**Helices (per 100 residues)**	**Average helix length (residues)**	**Strands (per 100 residues)**	**Average strand length (residues)**
**CONTINLL** [f]	6.3	15.7	0.8	2.0
**CDSSTR** [g]	4.8	17.5	1.3	4.2

[a] The 2Struc Secondary Structure Server (4) was used for secondary structure analysis from a PDB file.
[b] The structure of Rpn1 (PDB: 5MPC) was described previously (5).
[c] The STRuctural IDEntification method (6) uses hydrogen bond energies and phi-psi torsion angles to identify secondary structure.
[d] The Dictionary of Secondary Structure of Proteins (7) uses hydrogen bond energies to identify secondary structure.
[e] The DICHROWEB server (8) was used to analyze CD data.
[f] The CONTINLL deconvolution method was described previously (9). The normalized RMSD for this method was 0.062.
[g] The CDSSTR deconvolution method was described previously (10). The normalized RMSD for this method was 0.001.
[f,g] Deconvolution utilized reference protein data sets 4, 7, SP175, and SMP180 (11-13). Values for each type of secondary structure are averages across all reference sets.

Table S2: Photo-crosslinking sites identified in Ub^{T9Bpa}–Rpn1$^{214-355}$ after in-solution digestion.

Run 1 (denaturation with urea)						
**Crosslinked peptide (Rpn1$^{214-355}$–Ub^{T9Bpa})** [a]	**Crosslinked residue in Rpn1$^{214-355}$**	**Crosslinked residue in Ub** [b]	**Charge**	**Best score** [c]	**E-value** [d]	**No. of matches**
^{288}LGEEDMIR^{295}–^{7}TL*J*GK^{11}	M^{293}	T^{9Bpa}	2+	9.25E-01	3.25E-01	1
^{296}SVFDATSDPVMHK^{308}–^{7}TL*J*GK^{11}	S^{302}	T^{9Bpa}	2+, 3+	7.67-01	3.52E-02	2
^{309}QLAYILAAQK^{318}–^{7}TL*J*GK^{11}	Y^{312}	T^{9Bpa}	2+	9.57E-01	1.23E-02	2
^{345}ELNLTGPKPE^{352}–^{7}TL*J*GK^{11}	T^{349}	T^{9Bpa}	2+	3.29E-01	1.52E-02	3
Run 2 (denaturation with guanidine hydrochloride)						
**Crosslinked peptide (Rpn1$^{214-355}$–Ub^{T9Bpa})** [a]	**Crosslinked residue in Rpn1$^{214-355}$**	**Crosslinked residue in Ub** [b]	**Charge**	**Best score** [c]	**E-value** [d]	**No. of matches**
^{296}SVFDATSDPVMHK^{308}–^{7}TL*J*GK^{11}	T^{301}	T^{9Bpa}	5+	6.79E-02	9.26E-01	1
^{296}SVFDATSDPVMHK^{308}–^{7}TL*J*GK^{11}	M^{306}	T^{9Bpa}	3+, 4+	7.16E-01	2.23E-04	2
^{345}ELNLTGPKPE^{352}–^{7}TL*J*GK^{11}	L^{348}	T^{9Bpa}	4+	9.28E-01	4.20E-02	1

[a] Bpa is represented by *J* in the peptide sequence.
[b] T9Bpa is denoted here as T^{9Bpa}.
[c] Score values range from 0 to 1, where lower values correspond to greater probability of the match (14).
[d] E-values are the chances that the peptide score was achieved through incorrect matches from a database search.

Table S3: Photo-crosslinking sites identified in Ub^{T9Bpa}–$Rpn1^{214\text{-}324}$ after in-gel digestion.

Crosslinked peptide ($Rpn1^{214\text{-}324}$–Ub^{T9Bpa}) ^{a}	Crosslinked residue in $Rpn1^{214\text{-}324}$	Crosslinked residue in Ub ^{b}	Charge	Best score ^{c}	E-value ^{d}	No. of matches
^{233}LPQFVDENTFQR^{244}–^{7}TL*J*GK^{11}	L^{233}	T^{9Bpa}	4+	2.43E-5	5.65E-02	1
^{233}LPQFVDENTFQR^{244}–^{7}TL*J*GK^{11}	Q^{235}	T^{9Bpa}	4+	1.24E-1	3.95E-02	1
^{233}LPQFVDENTFQR^{244}–^{7}TL*J*GK^{11}	D^{238}	T^{9Bpa}	3+	2.10E-02	4.90E-03	1
^{233}LPQFVDENTFQR^{244}–^{7}TL*J*GK^{11}	T^{241}	T^{9Bpa}	4+	5.91E-02	2.36E-02	1
^{233}LPQFVDENTFQR^{244}–^{7}TL*J*GK^{11}	F^{242}	T^{9Bpa}	3+, 4+	3.02E-06	2.71E-04	2
^{288}LGEEDMIR^{295}–^{7}TL*J*GK^{11}	L^{288}	T^{9Bpa}	3+, 4+	9.68E-08	4.81E-04	5
^{288}LGEEDMIR^{295}–^{7}TL*J*GK^{11}	E^{291}	T^{9Bpa}	3+, 4+	9.46E-05	6.51E-5	7
^{288}LGEEDMIR^{295}–^{7}TL*J*GK^{11}	D^{292}	T^{9Bpa}	3+, 4+	2.10E-05	3.09E-05	11
^{288}LGEEDMIR^{295}–^{7}TL*J*GK^{11}	M^{293}	T^{9Bpa}	3+	2.68E-03	4.04E-04	2
^{296}SVFDATSDPVMHK^{308}–^{7}TL*J*GK^{11}	S^{296}	T^{9Bpa}	4+	1.10E-03	1.69E-02	1
^{296}SVFDATSDPVMHK^{308}–^{7}TL*J*GK^{11}	F^{298}	T^{9Bpa}	4+	7.64E-02	7.81E-01	1
^{296}SVFDATSDPVMHK^{308}–^{7}TL*J*GK^{11}	D^{299}	T^{9Bpa}	5+	2.37E-03	4.11E-01	1
^{296}SVFDATSDPVMHK^{308}–^{7}TL*J*GK^{11}	T^{301}	T^{9Bpa}	4+	4.26E-02	8.37E-02	2
^{296}SVFDATSDPVMHK^{308}–^{7}TL*J*GK^{11}	S^{302}	T^{9Bpa}	4+	1.65E-02	1.10E-01	2
^{296}SVFDATSDPVMHK^{308}–^{7}TL*J*GK^{11}	D^{303}	T^{9Bpa}	4+, 5+	2.57E-03	2.52E-02	2
^{296}SVFDATSDPVMHK^{308}–^{7}TL*J*GK^{11}	P^{304}	T^{9Bpa}	3+, 4+	7.54E-05	9.00E-03	5
^{309}QLAYILAAQK^{318}–^{7}TL*J*GK^{11}	Y^{312}	T^{9Bpa}	3+, 4+	7.28E-03	9.28E-03	5
^{309}QLAYILAAQK^{318}–^{7}TL*J*GK^{11}	I^{313}	T^{9Bpa}	3+, 4+	1.74E-04	3.35E-02	5
^{309}QLAYILAAQK^{318}–^{7}TL*J*GK^{11}	L^{314}	T^{9Bpa}	3+	1.43E-04	1.79E-02	1

^{a} Bpa is represented by *J* in the peptide sequence.
^{b} T9Bpa is denoted here as T^{9Bpa}.
^{c} Score values range from 0 to 1, where lower values correspond to greater probability of the match (14).
^{d} E-values are the chances that the peptide score was achieved through incorrect matches from a database search.

Table S4: Photo-crosslinking sites identified in K11-Ub_2^{Q49Bpa}–$Rpn1^{214\text{-}324}$ after in-gel digestion.

Crosslinked peptide ($Rpn1^{214\text{-}324}$–Ub^{T9Bpa}) [a]	Crosslinked residue in $Rpn1^{214\text{-}324}$	Crosslinked residue in Ub [b]	Charge	Best score [c]	E-value [d]	No. of matches
^{288}LGEEDMIR^{295}–^{49}*J*LEDGR^{54}	E^{291}	Q^{49Bpa}	3+	2.48E-01	8.43E-02	1
^{288}LGEEDMIR^{295}–^{49}*J*LEDGR^{54}	D^{292}	Q^{49Bpa}	3+	3.90E-02	1.82E-05	2
^{288}LGEEDMIR^{295}–^{49}*J*LEDGR^{54}	I^{294}	Q^{49Bpa}	4+	1.84E-02	1.55E-04	1
^{296}SVFDATSDPVMHK^{308}–^{49}*J*LEDGR^{54}	D^{303}	Q^{49Bpa}	4+	2.12E-01	3.01E-01	1
^{296}SVFDATSDPVMHK^{308}–^{49}*J*LEDGR^{54}	P^{304}	Q^{49Bpa}	4+	2.02E-03	2.46E-09	1

[a] Bpa is represented by *J* in the peptide sequence.
[b] Q49Bpa (denoted here as Q^{49Bpa}) is located in the proximal Ub of K11-Ub_2^{Q49Bpa}.
[c] Score values range from 0 to 1, where lower values correspond to greater probability of the match (14).
[d] E-values are the chances that the peptide score was achieved through incorrect matches from a database search

Table S5: Photo-crosslinking sites identified in K48-Ub$_2^{Q49Bpa}$–Rpn1$^{214\text{-}324}$ after in-gel digestion.

Crosslinked peptide (Rpn1$^{214\text{-}324}$–Ub^{T9Bpa}) [a]	Crosslinked residue in Rpn1$^{214\text{-}324}$	Crosslinked residue in Ub [b]	Charge	Best score [c]	E-value [d]	No. of matches
^{288}LGEEDMIR^{295}–^{49}*J*LEDGR^{54}	L^{288}	Q^{49Bpa}	3+, 4+	1.04E-03	5.69E-06	3
^{288}LGEEDMIR^{295}–^{49}*J*LEDGR^{54}	E^{291}	Q^{49Bpa}	3+	1.64E-02	1.14E-04	2
^{288}LGEEDMIR^{295}–^{49}*J*LEDGR^{54}	D^{292}	Q^{49Bpa}	3+	6.59E-03	1.97E-03	4
^{288}LGEEDMIR^{295}–^{49}*J*LEDGR^{54}	M^{293}	Q^{49Bpa}	3+	4.16E-05	1.81E-01	3
^{288}LGEEDMIR^{295}–^{49}*J*LEDGR^{54}	I^{294}	Q^{49Bpa}	3+, 4+	4.63E-02	3.78E-04	4
^{296}SVFDATSDPVMHK^{308}–^{49}*J*LEDGR^{54}	D^{303}	Q^{49Bpa}	4+	5.52E-03	4.14E-08	1
^{296}SVFDATSDPVMHK^{308}–^{49}*J*LEDGR^{54}	P^{304}	Q^{49Bpa}	3+	7.09E-02	1.49E-06	1
^{296}SVFDATSDPVMHK^{308}–^{49}*J*LEDGR^{54}	V^{305}	Q^{49Bpa}	4+, 5+	2.47E-01	7.29E-05	2

[a] Bpa is represented by *J* in the peptide sequence.
[b] Q49Bpa (denoted here as Q^{49Bpa}) is located in the proximal Ub of K48-Ub$_2^{Q49Bpa}$.
[c] Score values range from 0 to 1, where lower values correspond to greater probability of the match (14).
[d] E-values are the chances that the peptide score was achieved through incorrect matches from a database search.

Table S6: Photo-crosslinking sites identified in K63-Ub_2^{Q49Bpa}–$Rpn1^{214-324}$ after in-gel digestion.

Crosslinked peptide ($Rpn1^{214-324}$–Ub^{T9Bpa}) [a]	Crosslinked residue in $Rpn1^{214-324}$	Crosslinked residue in Ub [b]	Charge	Best score [c]	E-value [d]	No. of matches
^{288}LGEEDMIR^{295}–^{49}*J*LEDGR^{54}	M^{293}	Q^{49Bpa}	4+	2.79E-01	6.14E-04	1
^{288}LGEEDMIR^{295}–^{49}*J*LEDGR^{54}	I^{294}	Q^{49Bpa}	3+, 4+	4.05E-02	1.73E-03	2
^{296}SVFDATSDPVMHK^{308}–^{49}*J*LEDGR^{54}	D^{303}	Q^{49Bpa}	4+	2.59E-01	8.32E-03	1
^{296}SVFDATSDPVMHK^{308}–^{49}*J*LEDGR^{54}	P^{304}	Q^{49Bpa}	4+	3.20E-01	3.03E-02	1
^{296}SVFDATSDPVMHK^{308}–^{49}*J*LEDGR^{54}	M^{306}	Q^{49Bpa}	4+	2.81E-01	2.08E-05	1
^{296}SVFDATSDPVMHK^{308}–^{49}*J*LEDGR^{54}	H^{307}	Q^{49Bpa}	4+	1.22E-01	2.61E-07	1

[a] Bpa is represented by *J* in the peptide sequence.
[b] Q49Bpa (denoted here as Q^{49Bpa}) is located in the proximal Ub of K63-Ub_2^{Q49Bpa}.
[c] Score values range from 0 to 1, where lower values correspond to greater probability of the match (14).
[d] E-values are the chances that the peptide score is achieved through incorrect matches from a database search.

Supporting References

1. Sievers, F., Wilm, A., Dineen, D., Gibson, T. J., Karplus, K., Li, W., Lopez, R., McWilliam, H., Remmert, M., Söding, J., Thompson, J. D., and Higgins, D. G. (2011) Fast, scalable generation of high-quality protein multiple sequence alignments using Clustal Omega. *Mol Syst Biol* **7**, 539
2. Ryabov, Y., and Fushman, D. (2006) Interdomain Mobility in Di-Ubiquitin Revealed by NMR. *Proteins* **63**, 787-796
3. Shi, Y., Chen, X., Elsasser, S., Stocks, B. B., Tian, G., Lee, B. H., Zhang, N., de Poot, S. A., Tuebing, F., Sun, S., Vannoy, J., Tarasov, S. G., Engen, J. R., Finley, D., and Walters, K. J. (2016) Rpn1 provides adjacent receptor sites for substrate binding and deubiquitination by the proteasome. *Science* **351**, aad94211-aad942110
4. Klose, D. P., Wallace, B. A., and Janes, R. W. (2010) 2Struc: the secondary structure server. *Bioinformatics* **26**, 2624-2625
5. Wehmer, M., Rudack, T., Beck, F., Aufderheide, A., Pfeifer, G., Plitzko, J. M., Förster, F., Schulten, K., Baumeister, W., and Sakata, E. (2017) Structural insights into the functional cycle of the ATPase module of the 26S proteasome. *Proc Natl Acad Sci U S A* **114**, 1305-1310
6. Frishman, D., and Argos, P. (1995) Knowledge-based protein secondary structure assignment. *Proteins* **23**, 566-579
7. Kabsch, W., and Sander, C. (1983) Dictionary of protein secondary structure: pattern recognition of hydrogen-bonded and geometrical features. *Biopolymers* **22**, 2577-2637
8. Whitmore, L., and Wallace, B. A. (2004) DICHROWEB, an online server for protein secondary structure analyses from circular dichroism spectroscopic data. *Nucleic Acids Res* **32**, W668-673
9. van Stokkum, I. H., Spoelder, H. J., Bloemendal, M., van Grondelle, R., and Groen, F. C. (1990) Estimation of protein secondary structure and error analysis from circular dichroism spectra. *Anal Biochem* **191**, 110-118
10. Manavalan, P., and Johnson, W. C. (1987) Variable selection method improves the prediction of protein secondary structure from circular dichroism spectra. *Anal Biochem* **167**, 76-85
11. Sreerama, N., and Woody, R. W. (2000) Estimation of protein secondary structure from circular dichroism spectra: comparison of CONTIN, SELCON, and CDSSTR methods with an expanded reference set. *Anal Biochem* **287**, 252-260
12. Lees, J. G., Miles, A. J., Wien, F., and Wallace, B. A. (2006) A reference database for circular dichroism spectroscopy covering fold and secondary structure space. *Bioinformatics* **22**, 1955-1962
13. Abdul-Gader, A., Miles, A. J., and Wallace, B. A. (2011) A reference dataset for the analyses of membrane protein secondary structures and transmembrane residues using circular dichroism spectroscopy. *Bioinformatics* **27**, 1630-1636
14. Chen, Z. L., Meng, J. M., Cao, Y., Yin, J. L., Fang, R. Q., Fan, S. B., Liu, C., Zeng, W. F., Ding, Y. H., Tan, D., Wu, L., Zhou, W. J., Chi, H., Sun, R. X., Dong, M. Q., and He, S. M. (2019) A high-speed search engine pLink 2 with systematic evaluation for proteome-scale identification of cross-linked peptides. *Nat Commun* **10**, 3404
